# The hsa_circ_0000276-ceRNA regulatory network and immune infiltration in cervical cancer

**DOI:** 10.1186/s12885-023-10636-5

**Published:** 2023-03-09

**Authors:** Honglei Zhang, Xiuting Wang, Yaqin Li, Ying Bai, Qi Li, Shuling Wang, Yimiao Wei, Jiarong Li, Songquan Wen, Weihong Zhao

**Affiliations:** 1 Pathology and Pathophysiology Department, Basic Medical College, Shanxi Medical University, Taiyuan, 030001 China; 2 Biochemistry and Molecular Biology Department, Basic Medical College, Shanxi Medical University, Taiyuan, 030001 China; 3 The Second Clinical Medical College, Shanxi Medical University, Taiyuan, 030001 China; 4 Department of Epidemiology, School of Public Health, Shanxi Medical University, Taiyuan, 030001 China; 5 Department of Obstetrics and Gynecology, The Second Hospital of Shanxi Medical University, Taiyuan, 030001 China

**Keywords:** Cervical cancer, Human papillomavirus, Competing endogenous RNA, miR-154-5p, Immune infiltration, Prognosis

## Abstract

**Background:**

Our previous studies have confirmed that miR-154-5p can regulate pRb expression, and thus, play a tumor suppressor role in HPV16 E7-induced cervical cancer. However, its upstream molecules have not been elucidated in the progression of cervical cancer. This study aimed to explore the role of the miR-154-5p upstream molecule, hsa_circ_0000276 in cervical cancer development and its possible mechanisms of action.

**Methods:**

We detected differences in whole transcriptome expression profiles of cervical squamous carcinoma and tissues adjacent to cervical cancer tissues from patients using microarray technology to predict circular RNAs (circRNAs) with binding sites to miR-154-5p. Quantitative reverse transcription polymerase chain reaction (qRT-PCR) was used to detect the expression of hsa_circ_0000276 (which had the strongest binding capacity to miR-154 and was selected as the target molecule) in cervical cancer tissues, followed by in vitro functional assays. Downstream microRNAs (miRNAs) and mRNAs of hsa_circ_0000276 were identified using transcriptome microarray data and databases, while the protein–protein interaction networks were obtained using STRING. A competing endogenous RNA (ceRNA) network centered on hsa_circ_0000276 was constructed using Cytoscape and GO and KEGG databases. Abnormal expression and prognosis of critical downstream molecules were analyzed using gene databases and molecular experiments. qRT-PCR and western blot analysis was performed to verify the expression of candidate genes.

**Results:**

We identified 4,001 differentially expressed circRNAs between HPV16-positive cervical squamous carcinoma and benign cervical tissues and 760 circRNAs targeting miR-154-5p, including hsa_circ_0000276. hsa_circ_0000276 and miR-154-5p directly bound, and hsa_circ_0000276 was upregulated, in cervical precancerous lesions and cervical cancer tissues and cells. Silencing hsa_circ_0000276 inhibited G1/S transition and cell proliferation and promoted apoptosis in SiHa and CaSki cells. Bioinformatics analysis showed that the hsa_circ_0000276 ceRNA network included 17 miRNAs and seven mRNAs, and downstream molecules of hsa_circ_0000276 were upregulated in cervical cancer tissues. These downstream molecules were associated with a poor prognosis and affected cervical cancer-associated immune infiltration. Of these, expression of CD47, LDHA, PDIA3, and SLC16A1 was downregulated in sh_hsa_circ_0000276 cells.

**Conclusions:**

Our findings show that hsa_circ_0000276 exerts cancer-promoting effects in cervical cancer and is an underlying biomarker for cervical squamous cell carcinoma.

**Supplementary Information:**

The online version contains supplementary material available at 10.1186/s12885-023-10636-5.

## Background

According to the data of the International Agency for Research on Cancer, there were approximately 604,127 new cases of and 341,831 deaths caused by cervical cancer worldwide in 2020, accounting for 6.5% of the new cases and 7.7% of new deaths in women worldwide, respectively [1]. China had the highest number of cases of cervical cancer (106,430) and the second highest number of estimated deaths (47,739) due to cervical cancer, accounting for approximately 18% of the global cervical cancer burden [2]. According to the World Health Organization’s (WHO) Global Strategy for Accelerated Elimination of Cervical Cancer, reducing the mortality and incidence of cervical cancer in China is an important part of the global strategy.

**Fig. 1:**
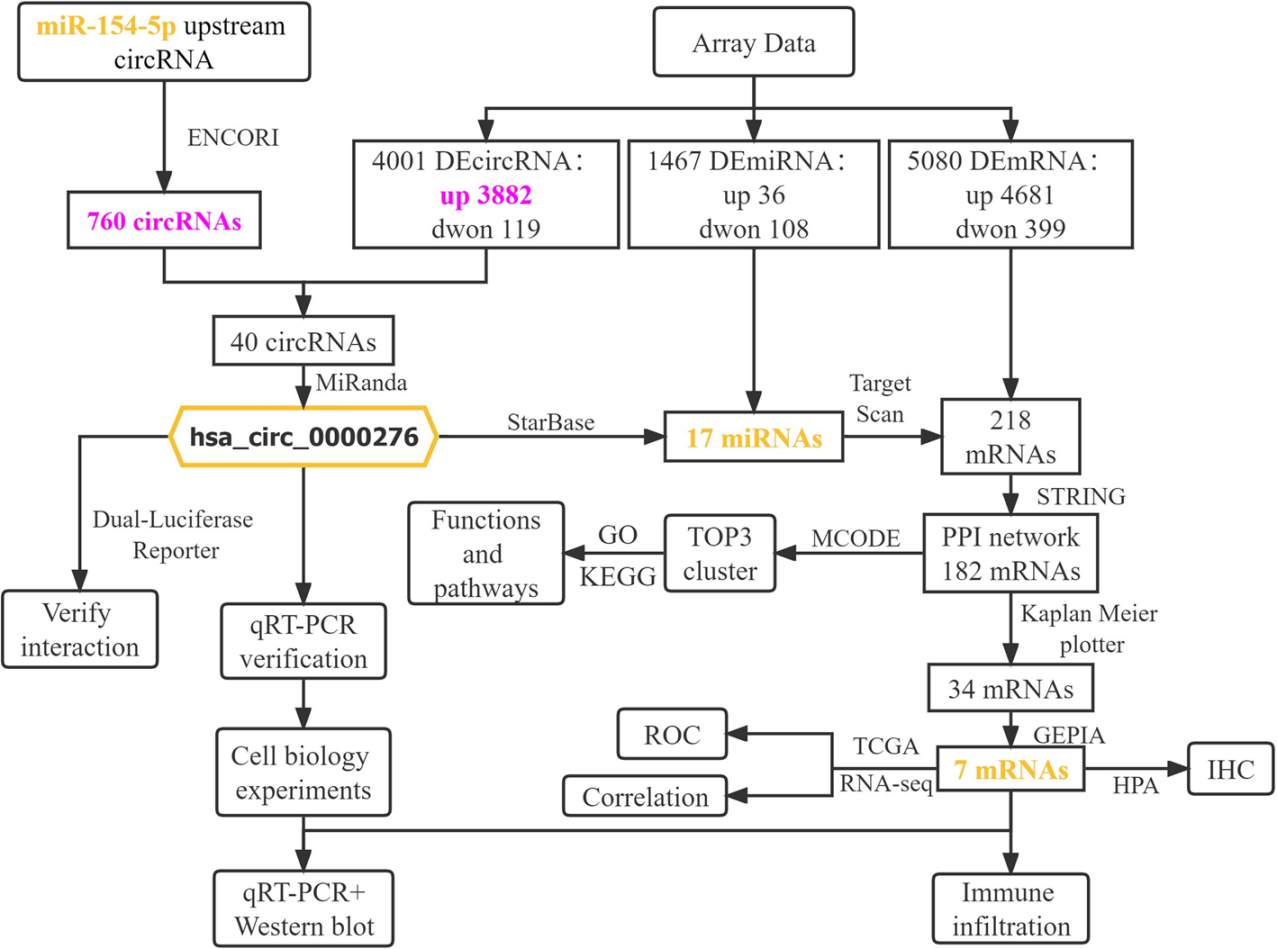
Research flow chart schematic of the analytic process

The occurrence of cervical cancer can be attributed to a combination of factors, and the underlying mechanism requires further investigation. The main causative factor of cervical cancer is persistent infection of high-risk human papillomavirus (HPV), with HPV16 being the most common type, accounting for more than 50% of cases. However, only 1% of those infected develop cervical cancer. Immune cells dominate the microenvironment of tumors, and the abilities of the immune cells play a crucial role in HPV infection. Although HPV vaccination has recently become popular worldwide, there is no specific treatment for women who are already infected with HPV or have progressed to cervical cancer. Therefore, continued understanding of the pathogenesis of cervical cancer is vital for its diagnosis, treatment, and prognosis. hsa-miR-154-5p was recently found to play a significant role in cancer. For example, MAPKAPK5-AS1 in hepatocellular carcinoma activates the epidermal growth factor receptor/Akt signaling pathway by sponging the microRNA (miRNA) miR-154-5p as a competing endogenous RNA (ceRNA), thereby upregulating the expression of PLAG1-like zinc finger 2 [3]. Exosome-derived miR-154-5p attenuates esophageal squamous cell carcinoma progression and angiogenesis by targeting Recombinant Kinesin Family-member 14 [4]. Knockdown of circABCC4 inhibits nuclear factor kappa B and Wnt/β-catenin signaling pathways, and upregulation of miR-154-5p inhibits the survival, migration, and invasion of breast cancer cells [5]. Evidently, miR-154-5p primarily plays an oncogenic role in tumors. Our previous study showed, for the first time, that miR-154-5p may play a tumor suppressor role in cervical carcinogenesis and that targeted regulation of Cullin2 inhibits the proliferation, invasion, and migration ability of HPV16-positive cervical cancer cells [6]. miR-154-5p has a powerful biological function in tumors; however, its upstream regulatory mechanism is unclear. ceRNA forms a regulatory network with miRNA at the core, and upstream noncoding RNAs (ncRNAs) competitively bind to miRNA response element. Recently, studies on upstream RNA have primarily focused on circular RNA (circRNA) or long ncRNA. circRNA is shaped by the reverse direction splicing of pre-mRNA. Unlike linear RNA, it does not contain the 5' and 3' terminal features to protect it from RNA exonuclease, and its expression is highly stable, with a circular covalently closed structure. Therefore, in some cases, circRNA may have higher expression levels than the corresponding linear RNAs [7]. circRNAs are involved in various cellular biological processes (BPs), from normal development to disease occurrence, and mediate cancer-related mechanisms, such as cell proliferation, autophagy, immune escape, and induction of angiogenesis. As ncRNAs, circRNAs regulate the expression of parent genes by interacting with miRNAs with binding sites; they can also affect the transcription of other genes and the formation and function of proteins. These findings indicate that the abnormality in the ceRNA network and mechanisms involved thereof, play a role in tumor formation. Considering this, when identifying ncRNAs upstream of miR-154-5p, we noted that hsa_circ_0000276 had the highest predicted score and lowest free energy for binding to miR-154-5p. Therefore, hsa_circ_0000276 was used as the starting point for the ceRNA network in this study. To the best of our knowledge, to date, no experimental studies on hsa_circ_0000276 have been conducted, and its role in cervical cancer remains unclear. Therefore, this study aimed to explore the impact and potential value of hsa_circ_0000276-miRNA-mRNA ceRNA in cervical carcinogenesis and identify new target molecules and potential regulatory mechanisms for the diagnosis and treatment of cervical cancer. A flowchart of our analysis is depicted in Fig. 1.

## Materials and methods

### Patients and tissue specimens

Seven tissue samples from of SCC and seven samples of healthy cervical tissue were used for microarray testing. Additionally, cervical tissue samples from 11 cases of chronic cervicitis, 16 cases of low-grade squamous intraepithelial lesion (LSIL), 20 cases of high-grade squamous intraepithelial lesion (HSIL), and 21 cases of cervical cancer were used to detect hsa_circ_0000276 expression. All samples were single HPV16-positive. From January 2019 to January 2022, samples were obtained from patients who underwent colposcopy at the Second Hospital of the Shanxi Medical University. The tissue specimens were independently diagnosed by two experienced clinical pathologists.

The inclusion criteria were as follows: (i) married women aged ≤ 65 years; (ii) residents of Taiyuan for > 1 year; and (iii) written informed consent provided. The exclusion criteria were as follows: (i) pregnant women; (ii) a history of hysterectomy; (iii) a history of treatment of cervical and vaginal diseases; and (iv) presence of other malignant tumors. Written informed consent was obtained from all study participants or their legal guardians. The Ethics Review Committee of the Second Hospital of the Shanxi Medical University approved this study [approval number (2019) YX No. (280)].

The operational protocol for cervical tissue collection was as follows. The doctor who performed the examination had > 2 years of experience. The lesion site was assessed, and if cervical cancer was considered, tissue was collected from: (1) the cancer site (two pieces of tissue [each approximately 5 mm in size] were clamped) and (2) the matching normal tissues (two pieces of tissues [each approximately 5 mm in size] were clamped at approximately 3–5 cm from the cancer site). After clamping, the tissues were placed in 10% neutral formalin for 6–12 h for fixation and processed within 24 h for routine pathological examination. The tissues were then collected in pre-cooled tubes and quickly snap frozen in liquid nitrogen.

### Microarray hybridization and data analysis

According to the Affymetrix GeneChip Expression Analysis Technical Manual (www.affymetrix.com) for performing microarray hybridization, data were analyzed using the default Affymetrix settings and the automated multi-chip analysis (robust multiarray analysis; RMA) algorithm. Values are shown as log2 RMA signal intensities. Differentially expressed (DE) mRNAs (DEMs), miRNAs (DEmiRNAs), and circRNAs (DEcircRNAs) in healthy and tumor tissues were identified using *p* < 0.05 and fold change (FC) > 1.2 as thresholds.

DEcircRNAs with upregulated expression in the microarray data were compared with those predicted to bind to miR-154-5p using Venny [8] in the Encyclopedia of RNA Interactomes (ENCORI) database [9]. Key circRNAs were identified using the miRanda database [10] based on the predicted score of each circRNA with miR-154-5p and binding free energy.

### Dual-luciferase reporter gene assay

Hsa_circ_0000276 wild-type (WT) and hsa_circ_0000276 mutant (MUT) gene fragments were designed and synthesized. The SacI and XhoI digestion sites were added to the two segments of the target fragment, which were then double-digested with the target fragment vector and cloned into the linearized GP-miRGLO vector. hsa-miR-154-5p mimics and mimic NC fragments were designed and synthesized according to the miR-154 gene sequence. The hsa_circ_0000276 WT sequence was constructed into the pmirGLO vector set; the hsa _circ_0000276 MUT sequence was constructed into the pmirGLO vector group; the hsa-miR-154-5p positive control (PC) was classified as the PC group; pmirGLO was the null group. HEK293T cells were incubated to 80–90% fusion and then inoculated in 12-well plates for 24 h. The vector and transfection reagents were added, and three replicate wells were established for each group. The transfection mixture was added and the cells were incubated for 5 h. The transfection solution was changed with DMEM containing fetal bovine serum for 24 h. The samples were collected and analyzed using a multifunctional enzyme marker, Infinite M1000 (Tecan, Männedorf, Switzerland), and fluorescence detection was performed.

### Cell culture and transfection

Cells were cultured in complete medium, composed of 10% fetal bovine serum (Sangong Biotech, Shanghai, China), 1% penicillin–streptomycin solution, and DMEM high-glucose medium, in a 5% CO_2_ incubator at 37 °C. The normal cervical epithelial cells HcerEpic and HPV16-positive human cervical squamous carcinoma SiHa and CaSki cells were grown in a culture dish, and the liquid was changed once every 2 days. Cells were transfected with short hairpin RNAs (shRNAs) designed to target hsa_circ_0000276. Cells were cultured in six-well plates, and when they reached approximately 70% confluence, they were transfected with shRNA or shNC (GenePharma, Shanghai, China) using Lipofectamine 2000 (Invitrogen, Waltham, USA) and collected after 24–48 h. Transfection efficiency in cells was verified using polymerase chain reaction (PCR).

### Extraction of total RNA

According to the manufacturer’s instructions, RNA fractions were isolated from the collected tissue or cell samples using a RNeasy Mini Kit (QIAGEN, Hilden, Germany). Tissues were sheared by immersing in lysis buffer and then crushed using a grinder. Ethanol was added to the lysate to provide suitable binding conditions, and the supernatant was transferred into a RNeasy Mini rotary column and washed. Lastly, 40 µL of RNase-free water was added to the column to elute the RNA, and a NanoDrop One spectrophotometer (Thermo Fisher Scientific, Waltham, USA) was used to assess the concentration and quality.

### Complementary DNA synthesis and real-time PCR

Reverse transcription was performed using the PrimeScriptTM RT Reagent Kit (Takara Bio, Kusatsu, Japan) under the following conditions: 60 min at 37 °C, 5 min at 85 °C, and storage at 4 °C. Next, 2 µL of the resulting complementary DNA was blended with the universal PCR mix and specific primers for hsa_circ_0000276 and seven mRNAs. Amplification was performed in triplicate using an Applied Biosystems 7500 Fast (Applied Biosystems, Waltham, USA) real-time fluorescent quantitative PCR system under the following thermocycling conditions: pre-denaturation at 95 °C for 30 s and PCR reaction for 40 cycles at 95 °C for 3 s and 60 °C for 30 s. Cycle threshold (Ct) values were calculated and recorded, and samples with invalid Ct values were excluded. Normalization was performed using 18S rRNA or GAPDH as internal reference controls. The primer sequences used are shown in Table 1.

**Table 1:** Primers for qPCR assays

Name	Primer sequence
18S-F	CCTGGATACCGCAGCTAGGA
18S-R	GCGGCGCAATACGAATGCCCC
GAPDH-F	CAGGAGGCATTGCTGATGAT
GAPDH-R	GAAGGCTGGGGCTCATTT
hsa_circ_0000276-F	TGCTCCCACAGATTGCTCCA
hsa_circ_0000276-R	AGGGAGCAGTGCAATGGATTT
CD47-F	AGTCTCTGTATTGCGGCGTG
CD47-R	GGGGTTCCTCTACAGCTTTCC
FKBP4-F	GAAGGCGTGCTGAAGGTCAT
FKBP4-R	GACAAAGACTCGGTCCCCAA
IRF-1-F	AAGCATGGCTGGGACATCAA
IRF-1-R	TGCTTTGTATCGGCCTGTGT
LDHA-F	TTGTCTCTGGCAAAGTGGAT
LDHA-R	ACCGCTTCCAATAACACGGT
PDIA3-F	CACGGACGACAACTTCGAGA
PDIA3-R	CTTCCCATCACGCGAGAACT
TFRC-F	GGCTACTTGGGCTATTGTAAAGG
TFRC-R	CAGTTTCTCCGACAACTTTCTCT
SLC16A1-F	GGTGGAGGTCCTATCAGCAGT
SLC16A1-R	CAGAAAGAAGCTGCAATCAAGC

### Cell counting kit-8 analysis

The proliferation rate of the SiHa and CaSki cells were determined using a Cell Counting Kit 8 (CCK-8) assay. The cells were cultured in 96-well plates for 24 h to reach a density of 3,000 cells/well. Then, 90 µL of serum-free medium and 10 µL of CCK-8 (Abxin, Shanghai, China) mixture were added to each well. After incubation with this mixture for 2 h, the absorbance was measured at 450 nm using a microplate reader.

### Cell cycle analysis

The SiHa and CaSki cell cycle were determined using the flow cytometer NovoCyte 3130 (ACEA Biosciences, San Diego, USA). Using a cell cycle detection kit (Yeasen Biotechnology, Shanghai, China), 1 × 10^6^ cells per sample were collected according to the manufacturer's instructions. Then, 70% ethanol was used for fixation at 4 °C for > 2 h, and cells were stained with propidium iodide then incubated for 30 min.

### Apoptosis analysis

SiHa and CaSki cell apoptosis were determined using a Cytomics FC500 Flow Cytometer (Beckman Coulter, Brea, USA). Detection was performed with an Annexin V-FITC/PI double-staining cell apoptosis detection kit (BD Biosciences, New Jersey, USA). According to the manufacturer's instructions, 1–5 × 10^5^ cells were collected from each sample. After staining, the cells were incubated in dark for 15 min, and apoptosis was detected using a computer.

### Western blotting analysis

Total protein was isolated from RIPA lysate and quantified using the BCA method. Next, 30 μg of total protein was separated by 8% sodium dodecyl sulfate polyacrylamide gel electrophoresis and transferred onto a polyvinylidene fluoride membrane. After blocking with 5% skim milk at 37 °C for 2 h, the membrane was incubated with primary antibodies against LDHA (1:1 000), CD47 (1:1 000), PDIA3 (1:1 000), SLC16A1 (1: 200), and the internal reference β-actin (1:1 000) overnight at 4 °C. The next day, the membrane was incubated with secondary antibodies at 37 °C for 1 h. The bands were detected by adding the chemiluminescence substrate, and proteins were quantified by densitometry using Image Lab 5.2 software, with β-actin as the internal reference. The experiment was performed three times.

### Establishment of the ceRNA network in SCC

Using the starBase database to predict hsa_circ_0000276 target miRNAs, TargetScan predicted the target genes of DEmiRNAs. The downregulated DEmiRNAs and upregulated DEMs were combined in the microarray data according to the mechanism of action of ceRNAs, integrating DEmiRNA-DEM and DEcircRNA-DEM co-expression as well as the resulting regulatory relationships. The circRNA-miRNA-mRNA network was constructed using Cytoscape.

### Construction of the protein–protein interaction network and MCODE

The protein–protein interaction (PPI) network was built and downloaded with the STRING database [11], and the top 10 hub genes were obtained using the CytoHubba [12] method after visualization with Cytoscape.

The mRNA groups with functional significance in cancer were clustered with the MCODE plug-in, according to the following criteria: MCODE score > 5, degree cut-off = 2, node score cut-off = 0.2, maximum depth = 100, and K-core = 2. We studied the biological functions and signaling mechanisms of the top three DEM clusters. Gene Ontology (GO) annotation included three aspects: BP, molecular function, and cellular component. The Kyoto Encyclopedia of Genes and Genomes (KEGG) was used to identify the possible pathways in which the molecule was involved, and the screening criterion was set at *p* < 0.05.

### Key mRNA analysis, expression, and enrichment

We assessed the prognostic value of DEmiRNAs using the Kaplan-Meier plotter database [13]. The Gene Expression Profiling Interactive Analysis (GEPIA) database [14] contains RNA microarray expression data for both tumor and healthy samples from The Cancer Genome Atlas (TCGA) and the Genome-Tissue Expression project. Target gene expression was analyzed using the GEPIA database with all parameters set to the default values to investigate the differential expression between tumor and healthy samples. Pathway analysis was performed to explore the role of these genes using the Metascape [15] online analysis tool.

### Statistical analysis

Statistical analysis was performed with SPSS 25.0 software (IBM, Armonk, USA), and visualization results were obtained with GraphPad Prism 8.0 software. The measurement data conforming to the normal distribution were expressed as the mean ± standard deviation. The *t*-test was used for comparisons between two groups, whereas the Kruskal -Wallis test followed by one-way analysis of variance (ANOVA) was used to compare differences between multiple groups. Statistical significance was set at *p* < 0.05.

## Results

### DEcircRNAs targeting miR-154-5p

**Fig. 2:**
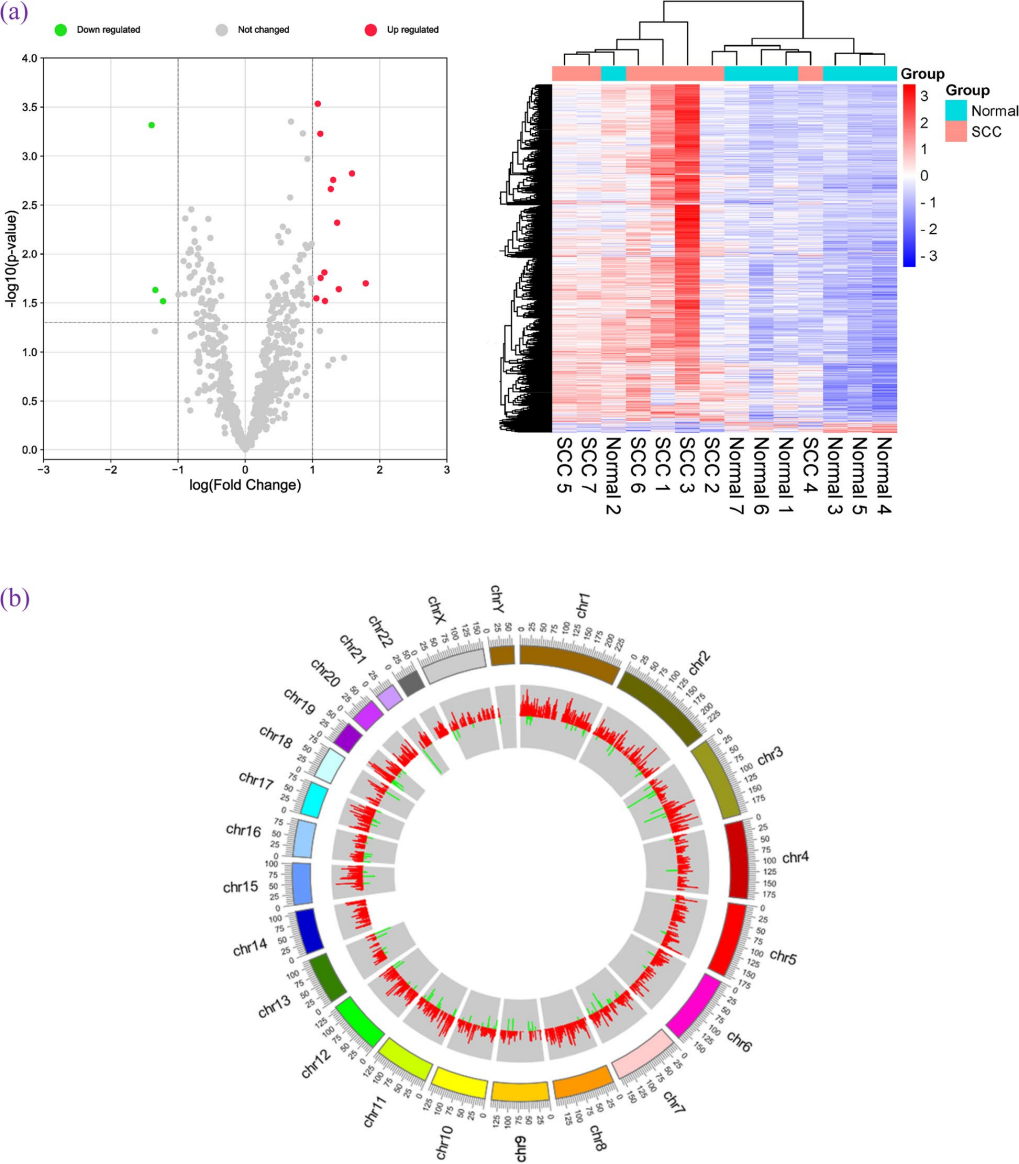
Differential expression of circRNA in cervical cancer. **a** Volcano plot and heat maps of the DEcircRNAs. **b** Distribution and expression of circRNA on human chromosomes

To investigate the effect of circRNA-associated ceRNAs in cervical cancer progression, we performed RNA microarray analysis in seven clinical cervical cancer tissues and their paired paracancerous normal tissues. A total of 4,001 circRNAs were detected, including 3,882 upregulated circRNAs and 119 downregulated circRNAs (Fig. 2a). The Circos graph shows the distribution and expression of the detected and significantly expressed circRNAs on human chromosomes (Fig. 2b).

**Fig. 3:**
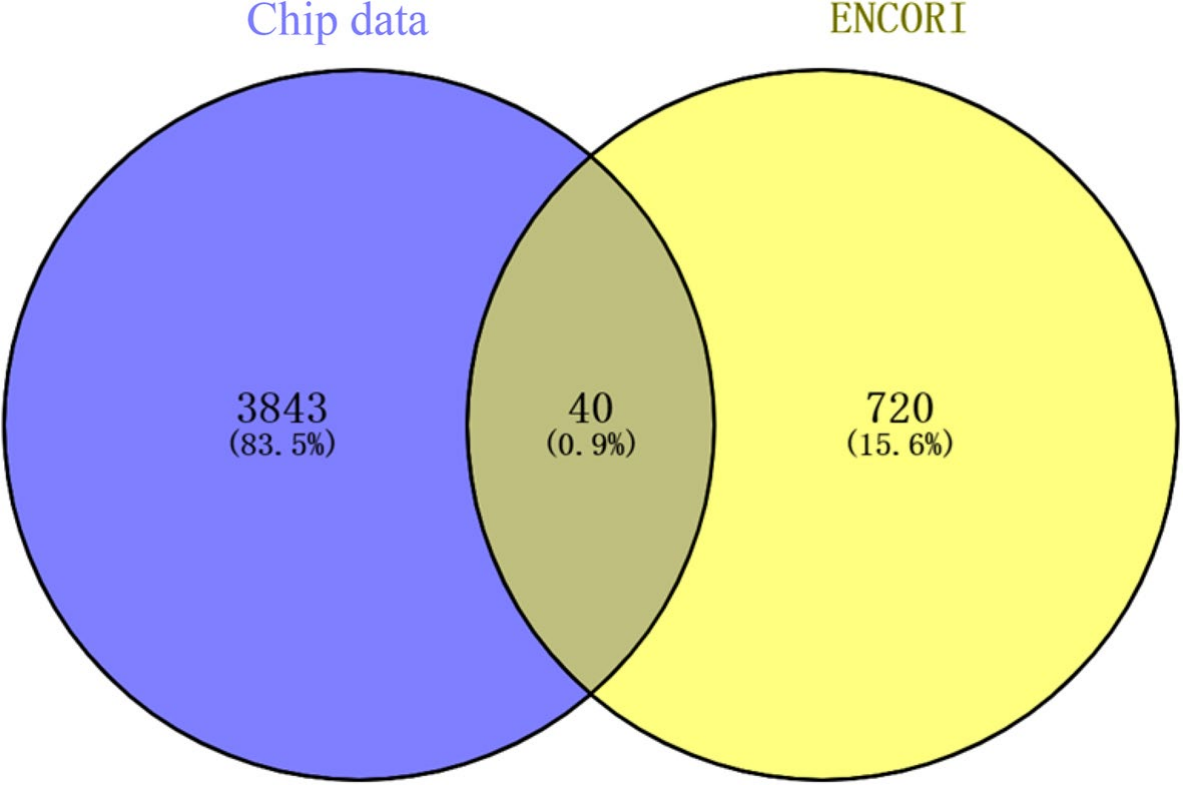
Common upregulated DEcircRNAs and circRNAs targeting miR-154-5p

The ENCORI database was used to forecast circRNAs upstream of miR-154-5p. We obtained 760 circRNAs and compared them with the circRNAs upregulated in cancer (FC ≥ 1.2) shown in chip information and 40 circRNAs at intersections (Fig. 3), of which 28 circRNAs had two or more binding sites with miR-154-5p and were upregulated in the chip data. miRanda software was used to calculate the predicted score and free energy of their binding to miR-154-5p. Additional File 1 shows the 28 circRNAs and their expression trends on the chip. circRNAs were ranked according to their predicted values. Among them, hsa_circ_0000276 (score = 167, energy =  − 27.76) was ranked first because of the high predicted value and lowest free energy, indicating that the binding probability was high and the most stable.

### miR-154-5p is the targeted miRNA of hsa_circ_0000276

**Fig. 4:**
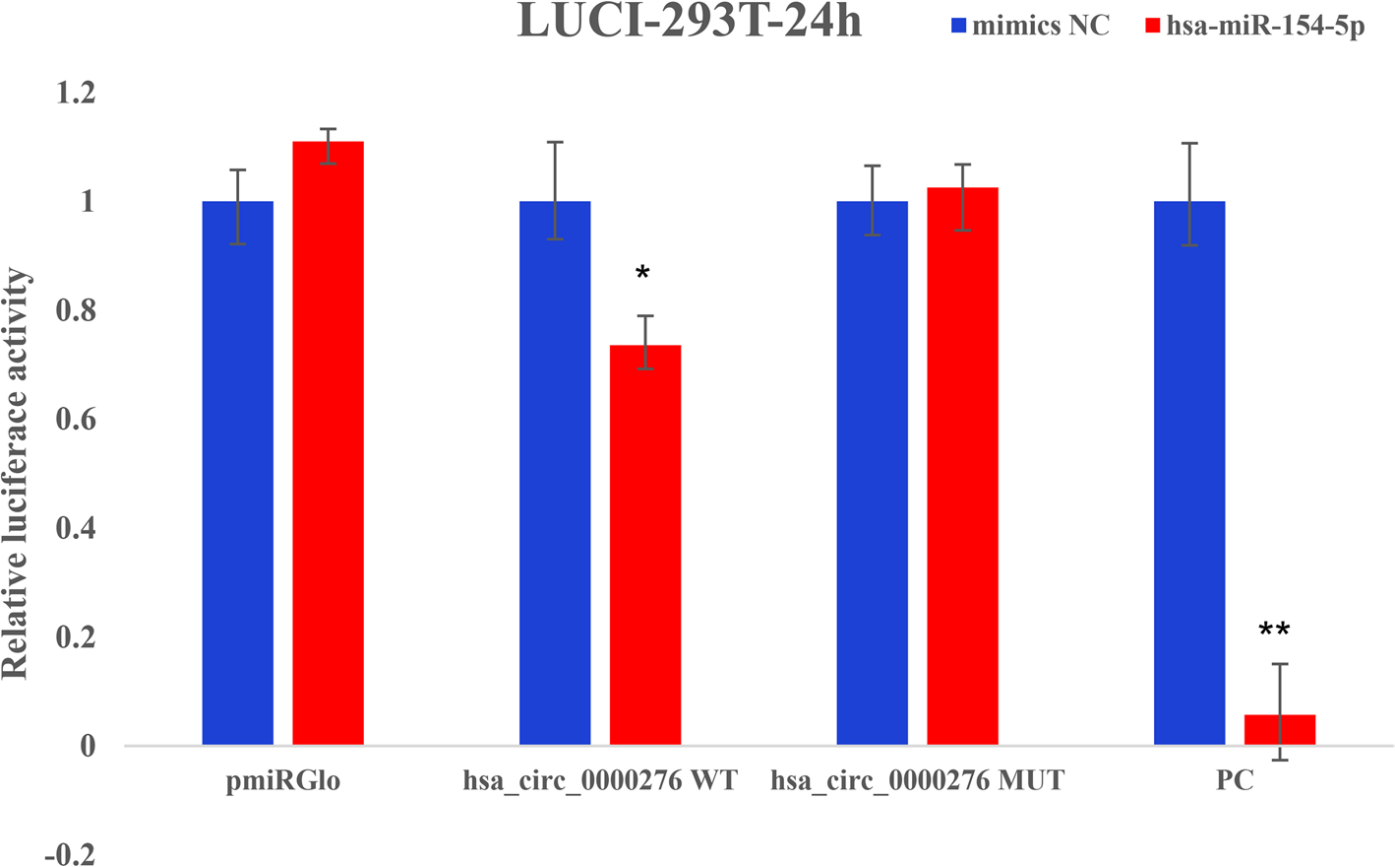
Dual-luciferase reporter gene assay of hsa_circ_0000276 and miR-154-5p, *p < 0.05, **p < 0.01

The luciferase activity of cells in the hsa_circ_0000276 WT group was significantly inhibited by the binding of miR-154-5p (*p* < 0.05) compared to that in the control group, while that in the hsa_circ_0000276 MUT group was not significantly different from that in the control group (*p* > 0.05; Fig. 4). Therefore, hsa_circ_0000276 has a direct binding effect with miR-154-5p.

### hsa_circ_0000276 is upregulated in cervical cancer tissues

**Fig. 5:**
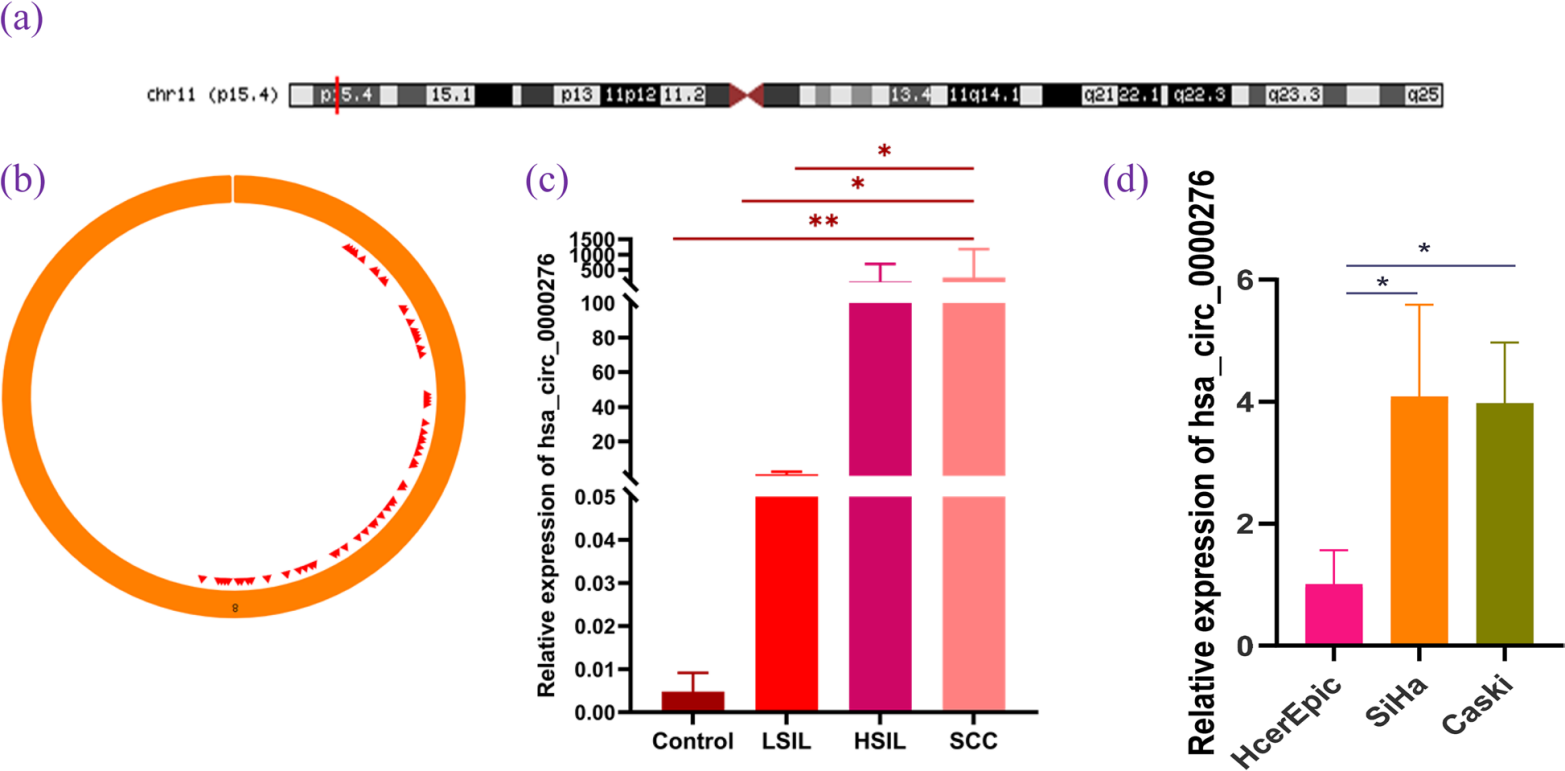
Structure of hsa_circ_0000276 and high expression in cervical cancer. **a** The Circbase database displays the location map of hsa_circ_0000276. **b** The CSCD database shows that circ_0000276 contains the MRE structure. **c** qRT-PCR verified the high expression of hsa_circ_0000276 in SCC. **d** qRT-PCR showed the high expression of hsa_circ_0000276 in cervical cancer cell lines. Data are presented as the mean ± SEM (*n* = 3 per group). *p < 0.05, **p < 0.01

Hsa_circ_0000276 is a 6,472 bp-long circRNA. Results from the Circbase database [16] show that circ_0000276 originates between exons three and four of the TRIM22 gene, also known as circTRIM22, on Chr11:5,711,031–5, 717,503 (Fig. 5a–b). The Cancer Specific CircRNA Database [17] showed that hsa_circ_0000276 contains an miRNA response element (MRE) structure, suggesting that hsa_circ_0000276 may be a key circRNA that regulates cervical carcinogenesis by sponging miRNAs.

To verify whether hsa_circ_0000276 is involved in HPV16-positive cervical carcinogenesis, we collected 68 samples of different HPV16-positive cervical tissues and defined chronic cervicitis tissues as the control group. Quantitative reverse-transcription PCR (qRT-PCR) was performed to detect hsa_circ_0000276 expression, and hsa_circ_0000276 expression was found to be significantly upregulated in the SCC group compared with that in the control (*p* = 0.002), HSIL (*p* = 0.047), and LSIL (*p* = 0.049) groups, while there was no significant difference between the LSIL, HSIL, and control groups (Fig. 5c). In addition, the in vitro validation results showed that the expression of hsa_circ_0000276 was higher in SiHa and CaSki cells than in HcerEpic cells, as shown in Fig. 5d (*p* < 0.05). These findings suggest that hsa_circ_0000276 may be a potential biomarker in HPV type 16 cervical cancer, which provides a partial basis for our subsequent experiments.

### sh-hsa_circ_0000276 inhibits the proliferation and promotes the apoptosis of cervical cancer cells

**Fig. 6:**
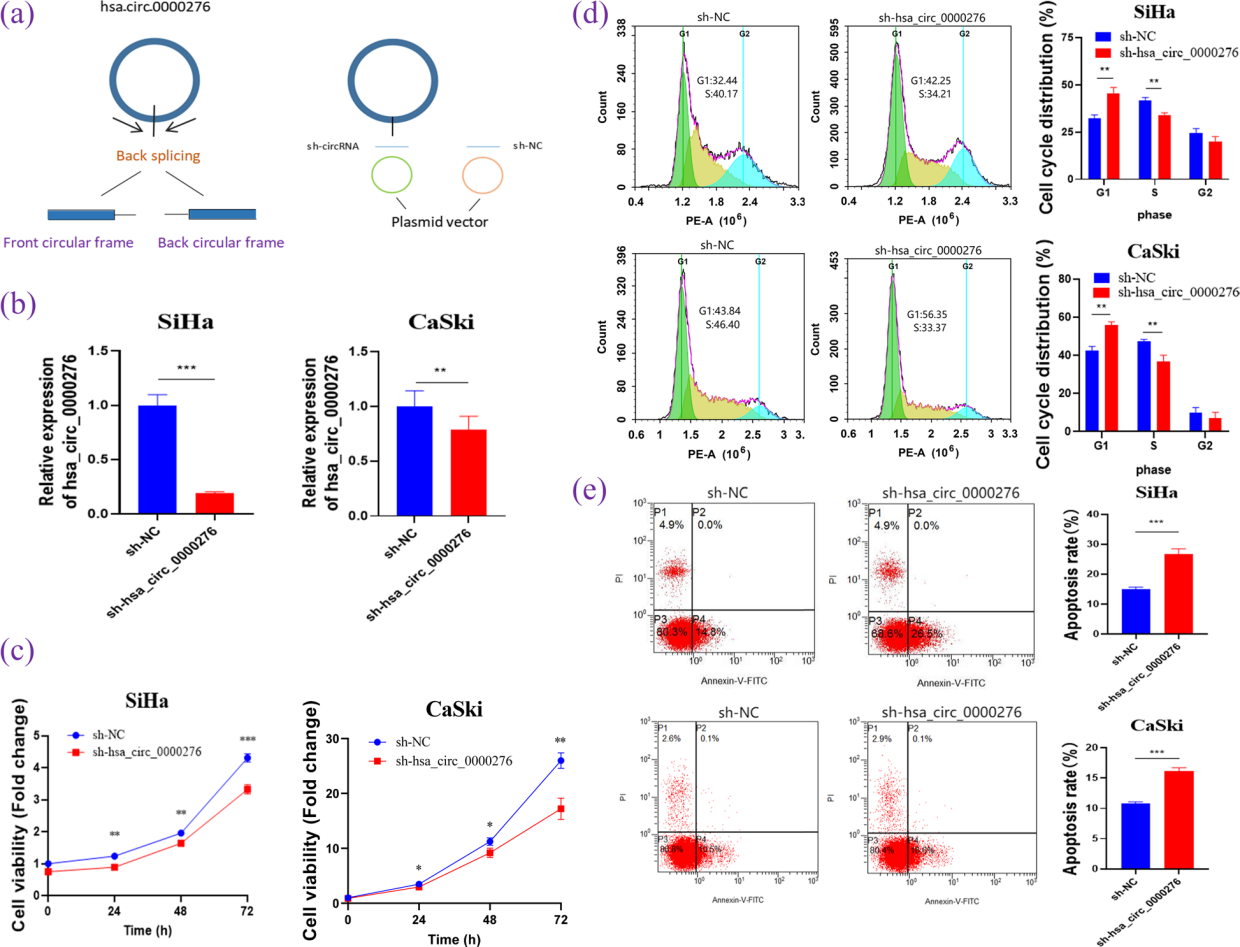
Silencing of hsa_circ_0000276 affects proliferation, cell cycle, and apoptosis of SiHa cells. **a** Schematic diagram of hsa_circ_0000276 and shRNA. **b** qRT-PCR analysis of the expression of hsa_circ_0000276 in SiHa and CaSki cells transfected with sh-NC or sh-hsa_circ_0000276. **c** Analysis of SiHa and CaSki cell growth curves by the CCK-8 method. **d** Flow cytometry analysis of SiHa and CaSki cell cycles. **e** Flow cytometry analysis for SiHa and CaSki cell apoptosis. Data are presented as the mean ± SEM (*n* = 3 per group). *p < 0.05, **p < 0.01, ***p < 0.001

As shown in Fig. 6a, shRNA targeting the hsa_circ_0000276 junction site was designed and transfected into SiHa and CaSki cells. qRT-PCR showed that the expression of hsa_circ_0000276 was significantly downregulated in SiHa (*p* < 0.001) and CaSki (*p* < 0.01) cells transfected with shRNA compared with that in the controls (Fig. 6b). In the CCK-8 proliferation assay (Fig. 6c), the growth curve showed that knockdown hsa_circ_0000276 significantly inhibited the proliferation of SiHa and CaSki cells compared with that of control cells. Cell cycle analysis (Fig. 6d) revealed that after silencing hsa_circ_0000276, more SiHa and Caski cells stagnated in the G1 phase, while there were fewer cells in the S phase, compared with that in the control group, indicating that SiHa and CaSki cells were arrested in the G1 phase (*p* < 0.01). As shown in Fig. 6e, the apoptosis rate of SiHa and CaSki cells increased significantly after silencing hsa_circ_0000276 compared with that in the control, indicating that silencing hsa_circ_0000276 promoted apoptosis (*p* < 0.001).

### Construction of basic DEM and miRNA relationship pairs

**Fig. 7:**
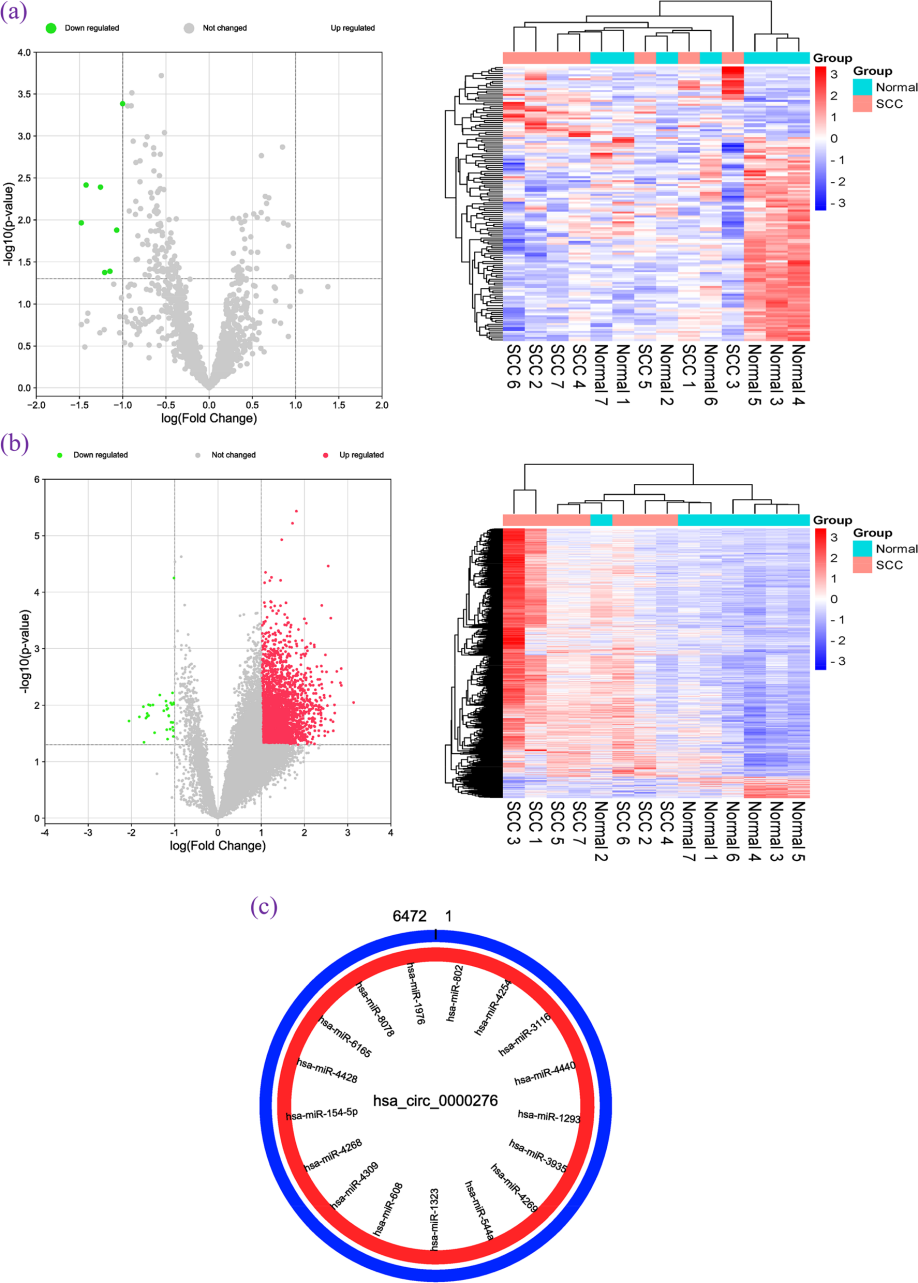
Screening of differentially expressed genes. (a-b) Volcano plot and heat maps of the array data. **a** miRNAs, (**b**) mRNAs, and (**c**) adsorption diagram of miRNAs and hsa_ circ_0000276

In the RNA microarray data, we identified 1,467 DEmiRNAs, with 36 upregulated miRNAs and 108 downregulated miRNAs, and 5,080 DEmRNAs, with 4,681 upregulated genes and 399 downregulated genes. Volcano and clustering heat maps (Fig. 7a–b) showed that miRNA and mRNA expression, respectively, were significantly abnormal in SCC tissues compared with that in matched healthy tissues adjacent to cancer (|FC|> 1.2, *p* < 0.05). Using the hsa_circ_0000276-targeted miRNAs predicted and obtained from the starBase database, compared with miRNAs displaying downregulated expression in the microarray data, we identified 17 miRNAs with a potential binding effect with hsa_circ_0000276. The adsorption map with hsa_circ_0000276 was drawn according to the starting coordinates and lengths of the miRNAs (Fig. 7c).

**Fig. 8:**
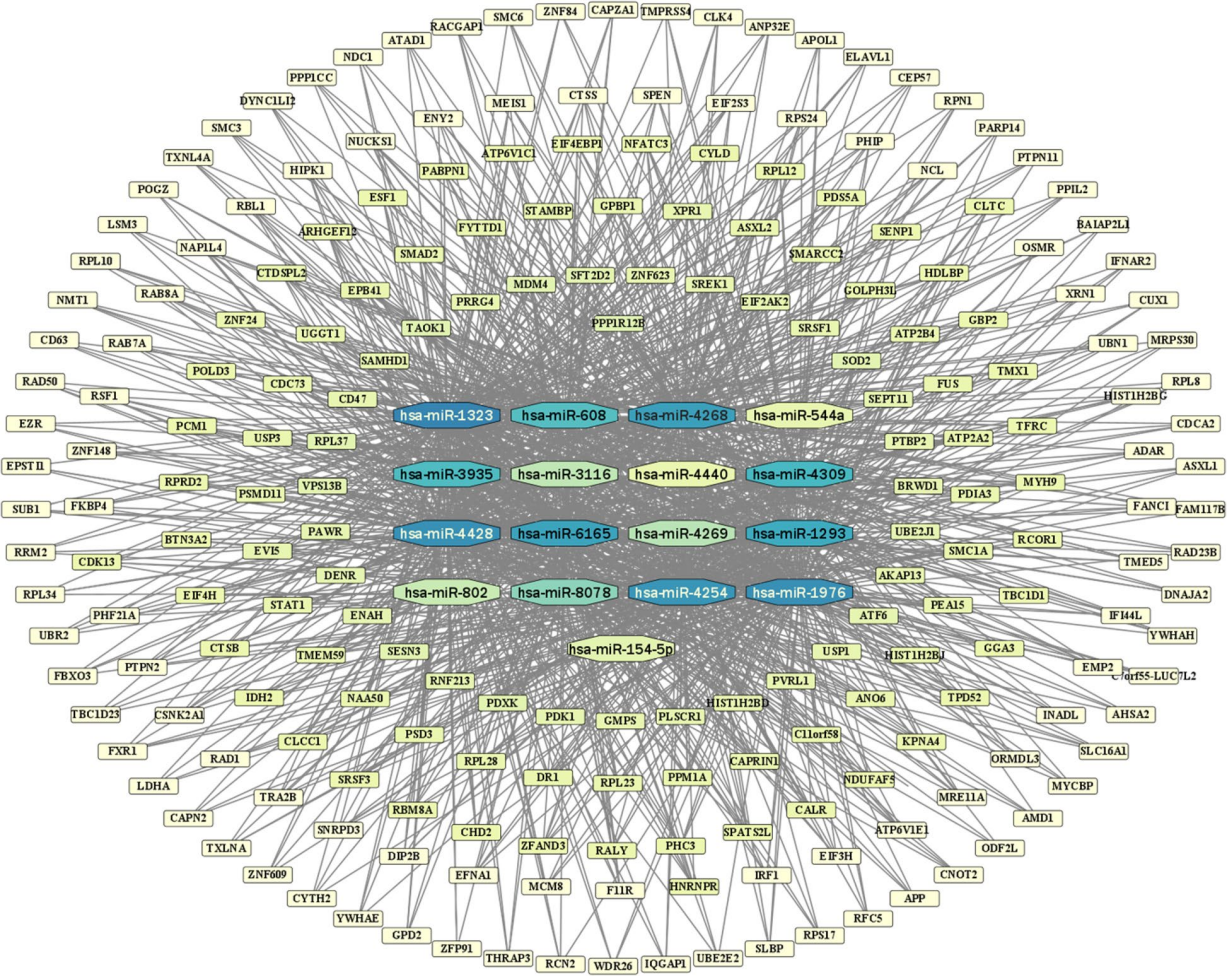
Basic DEM and miRNA relationship pairs. (Rectangles represent mRNAs, hexagons represent miRNAs, and the depth of color represents the size of the Degree value of the molecule in the whole relationship pair; namely, the darker the color, the more lines the molecule connects in the diagram)

The DEmiRNAs were uploaded to the TargetScan database. Combined with the highly expressed (FC ≥ 3) DEMs shown in the microarray data in cervical cancer, the basic DEM-miRNA relationship pairs in the network were identified using Cytoscape visualization. After discarding the DEMs with FC < 3, 218 DEMs were obtained. Figure 8 displays the DEMs in concentric circles; the closer a molecule is to the center of the circle, the more connected it is to the DEmiRNAs.

### PPI, biological functions, and signaling pathways involved in the ceRNA network mechanism

We uploaded the 218 screened mRNAs to the STRING database for further development of the PPI network. Subsequently, 182 mRNAs were filtered into the PPI network, which contained 182 nodes and 630 edges. The large network was clustered with the MCODE plug-in through topology to identify densely connected regions. The clustering modules with the top three highest calculated scores were subjected to GO and KEGG analyses.

**Fig. 9:**
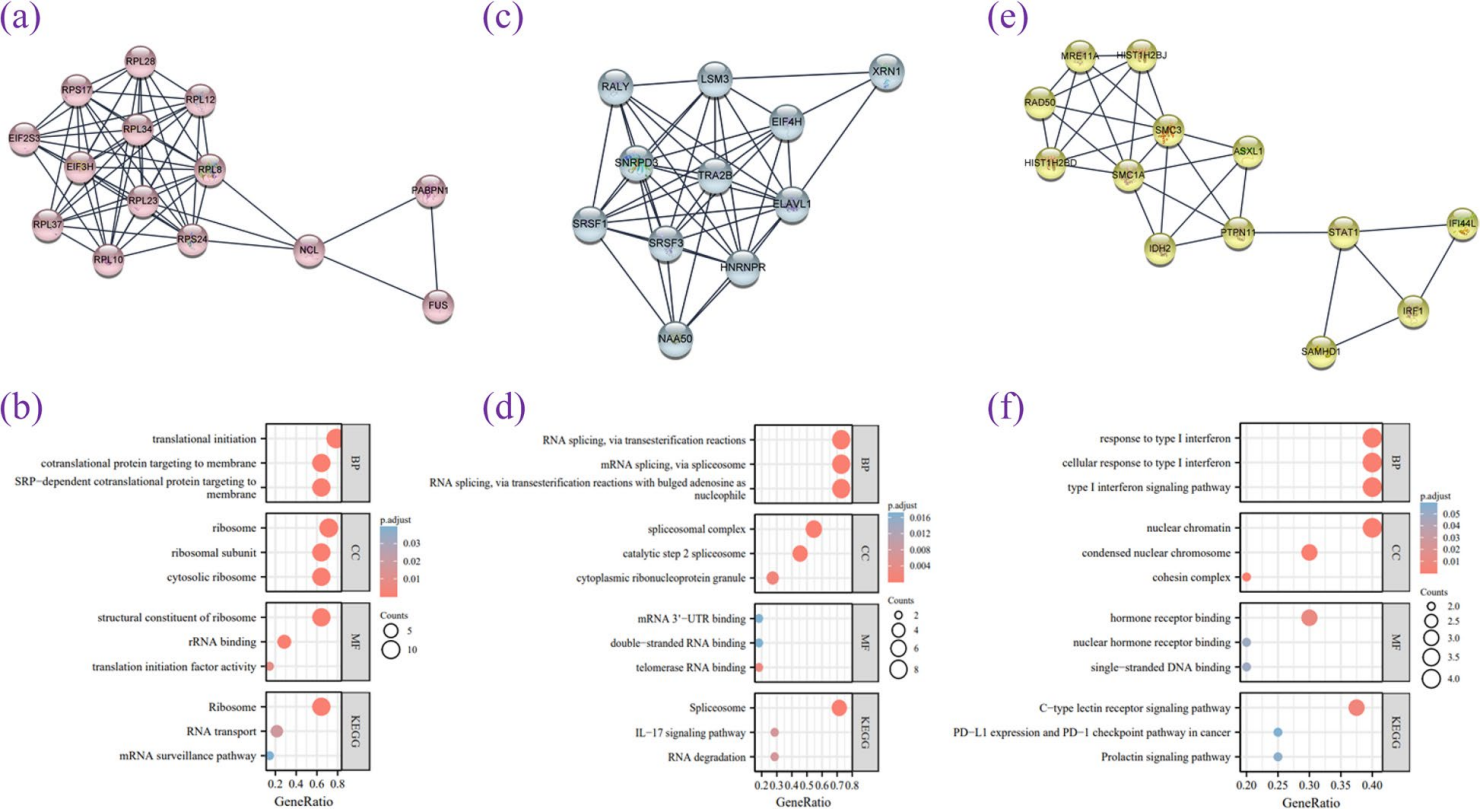
Results of MCODE analysis in common DEMs. **a**,**c**,**e** The Cytoscape plugin MCODE was used to identify the important modules in DEGs, and then the final three important modules were filtered according to the filtering criteria. **b**,**d**,**f** Results of the three important modules pathway enrichment analyses

Clustering module 1 contained 14 nodes and 60 edges, and the genes in module 1 were primarily related to translation initiation, RNA transport, and ribosomes (Fig. 9a–b). Cluster module 2 contained 11 nodes and 44 edges. The genes in module 2 were mainly associated with mRNA 3' untranslated region binding, RNA degradation, mRNA splicing via spliceosomes, and the interleukin 17 signaling pathway (Fig. 9c–d). Cluster module 3 contained 13 nodes and 30 edges. The genes in this module were primarily related to nuclear chromatin, single-stranded DNA binding, programmed cell death (PD) ligand 1 (PD-L1) expression, and the PD-1 checkpoint pathway in cancer (Fig. 9f–g).

### Screening and verification of prognostic genes in the ceRNA network using Kaplan–Meier survival analysis database

**Fig. 10:**
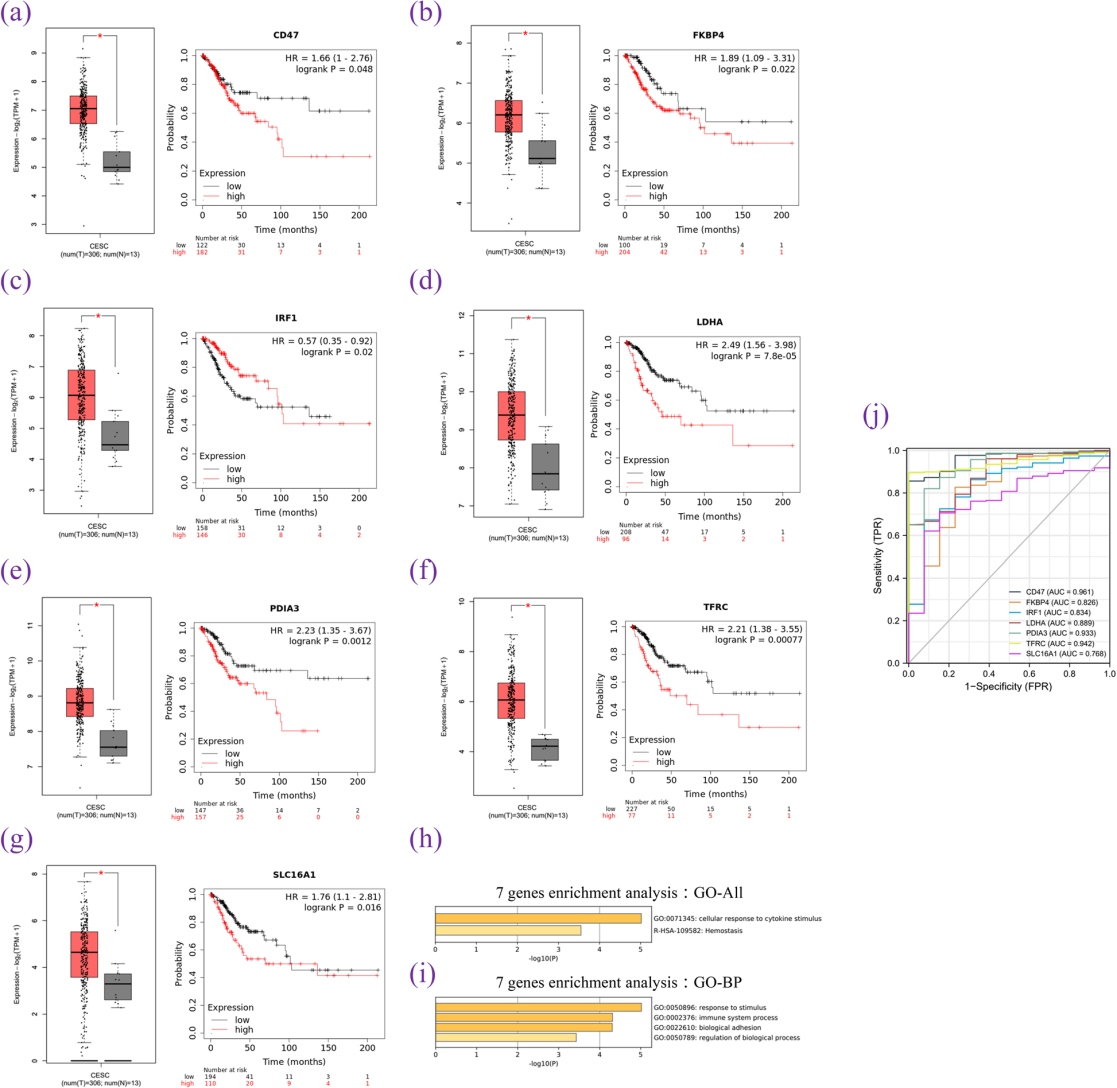
Expression and survival analysis of seven DEMs. **a**-**g** Expression verification based on the GEPIA database and survival analysis based on the Kaplan-Meier plotter database. **h** Bar graph of enriched terms across input gene lists. **i** The top-level biological processes can be enriched. **j** ROC analysis of the seven core mRNAs in CC

The prognostic value of the expression of 182 mRNAs in the PPI network was evaluated with the Kaplan–Meier database. The results showed that 34 genes significantly influenced the prognosis of cervical cancer, and a poor prognosis of cervical cancer was relevant to the high expression of these genes. These genes were uploaded to the GEPIA database (Fig. 10a–g), which showed that in cervical cancer and paracancerous tissues, the expression levels of seven genes, CD47, FKBP4, IRF-1, LDHA, PDIA3, TFRC, and SLC16A1, were significantly higher in cancer tissues (*p* < 0.05) than in healthy tissues. Using the default settings in the Metascape database, we found that the seven genes in the BP group were associated with the cellular response to cytokine stimulus, which was mainly concentrated on immune system processes and biological adhesion (Fig. 10h–i).

The area under the receiver operating characteristic curve (AUC) of the receiver operating characteristic (ROC) curve was used to evaluate the predictive ability of the genes for cervical cancer (Fig. 10j). We found that the accuracy of the predictive ability of the seven key mRNAs was very high. CD47 [AUC = 0.961, confidence interval (CI) = 0.931–0.991], PDIA3 (AUC = 0.933, CI = 0.876–0.990), and TFRC (AUC = 0.942, CI = 0.913–0.970) had a high accuracy of prediction. FKBP4 (AUC = 0.826, CI = 0.699–0.953), IRF-1 (AUC = 0.834, CI = 0.726–0.942), LDHA (AUC = 0.889, CI = 0.813–0.965), and SLC16A1 (AUC = 0.768, CI = 0.662–0.875) had a certain prediction accuracy.

### Key mRNA interactions and high expression in SCC

**Fig. 11:**
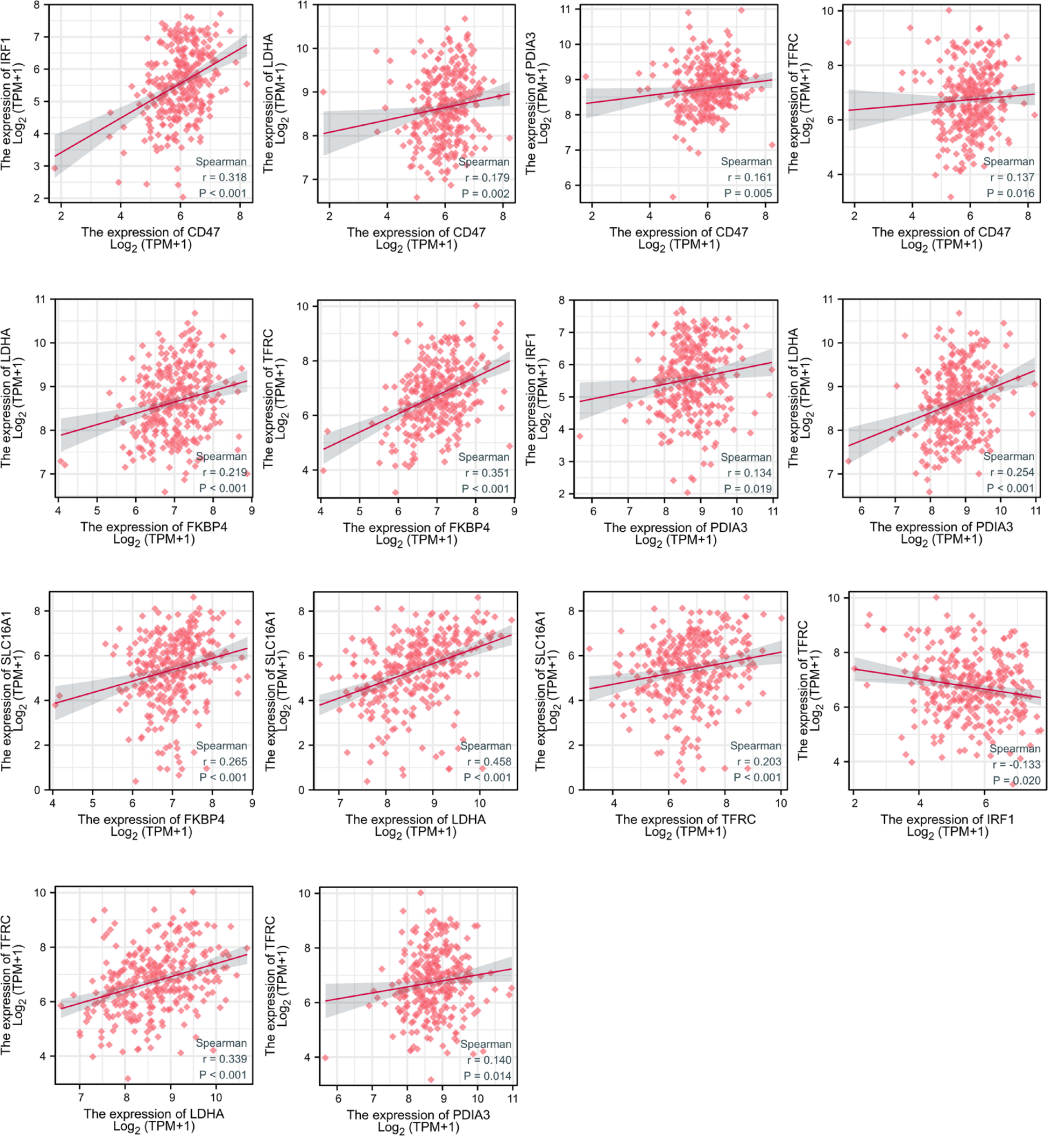
Correlation of seven mRNA expressions

In this study, common target genes based on hsa_circ_0000276 targeting miRNAs were investigated. Seven key genes were identified, and all seven were found to be upregulated in cervical cancer. To further observe the correlation between the key genes and hsa_circ_0000276, we used the RNA microarray data for cervical cancer in TCGA. We found that among the molecules demonstrating correlation, excluding IRF-1 and TFRC, expression in cervical cancer was negatively correlated with hsa_circ_0000276 expression, and expression of the key genes was positively correlated with one another (Fig. 11).

**Fig. 12:**
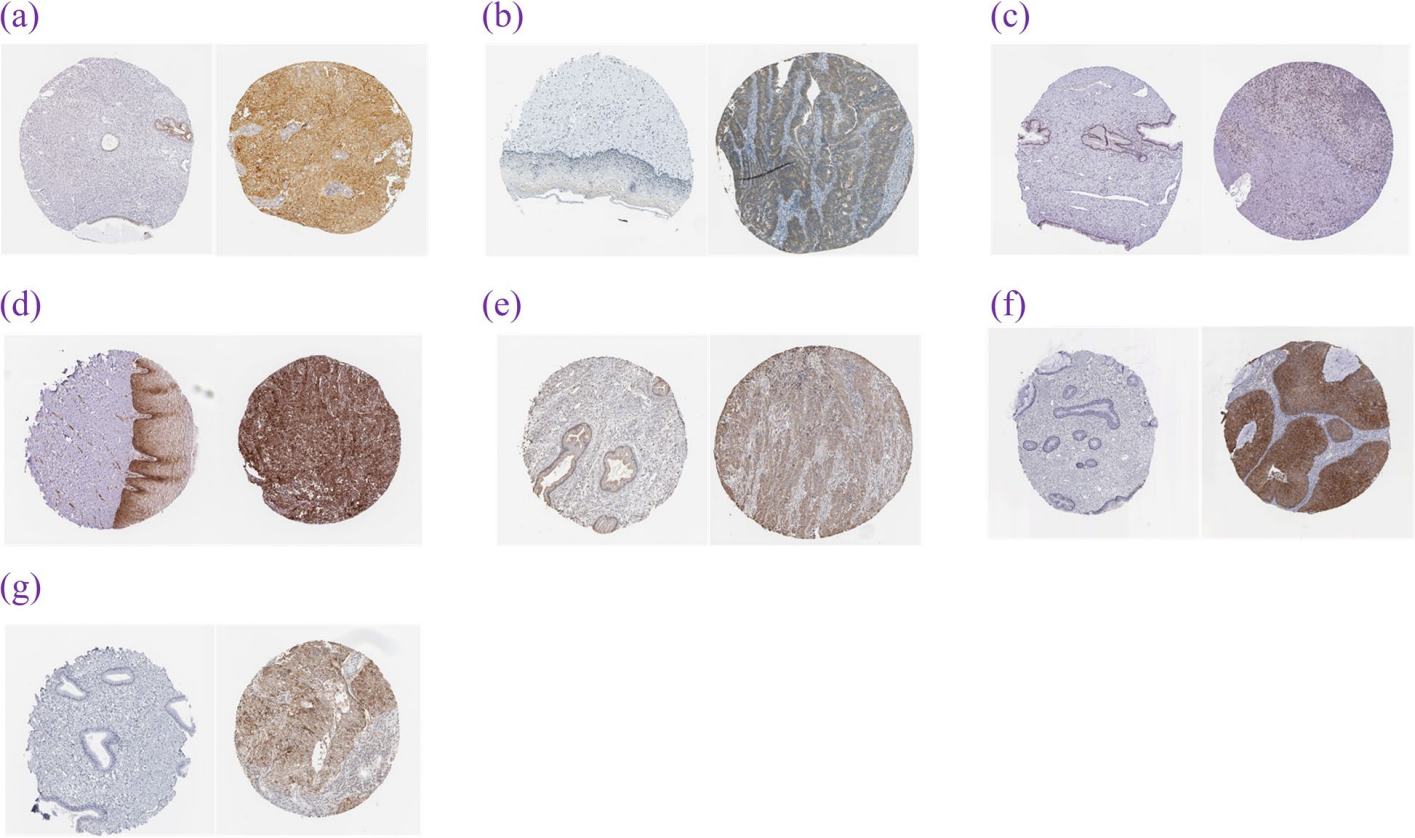
Immunohistochemistry sections of normal tissues and cervical cancer in the Human Protein Atlas. **a** CD47, (**b**) FKBP4, (**c**) IRF-1, (**d**) LDHA, (**e**) PDIA3, (**f**) TFRC, and (**g**) SLC16A1

The Human Protein Atlas database [18] showed differences in the expression of CD47, FKBP4, IRF-1, LDHA, PDIA3, TFRC, and SLC16A1 in healthy and cervical cancer tissues by immunohistochemistry, and the results of antigen-specific recognition in tissues using the same antibody codes are shown in Fig. 12a–g. These differences in staining intensity verified our hypothesis.

### mRNAs in immunocyte-enriched/depleted SCC

**Fig. 13:**
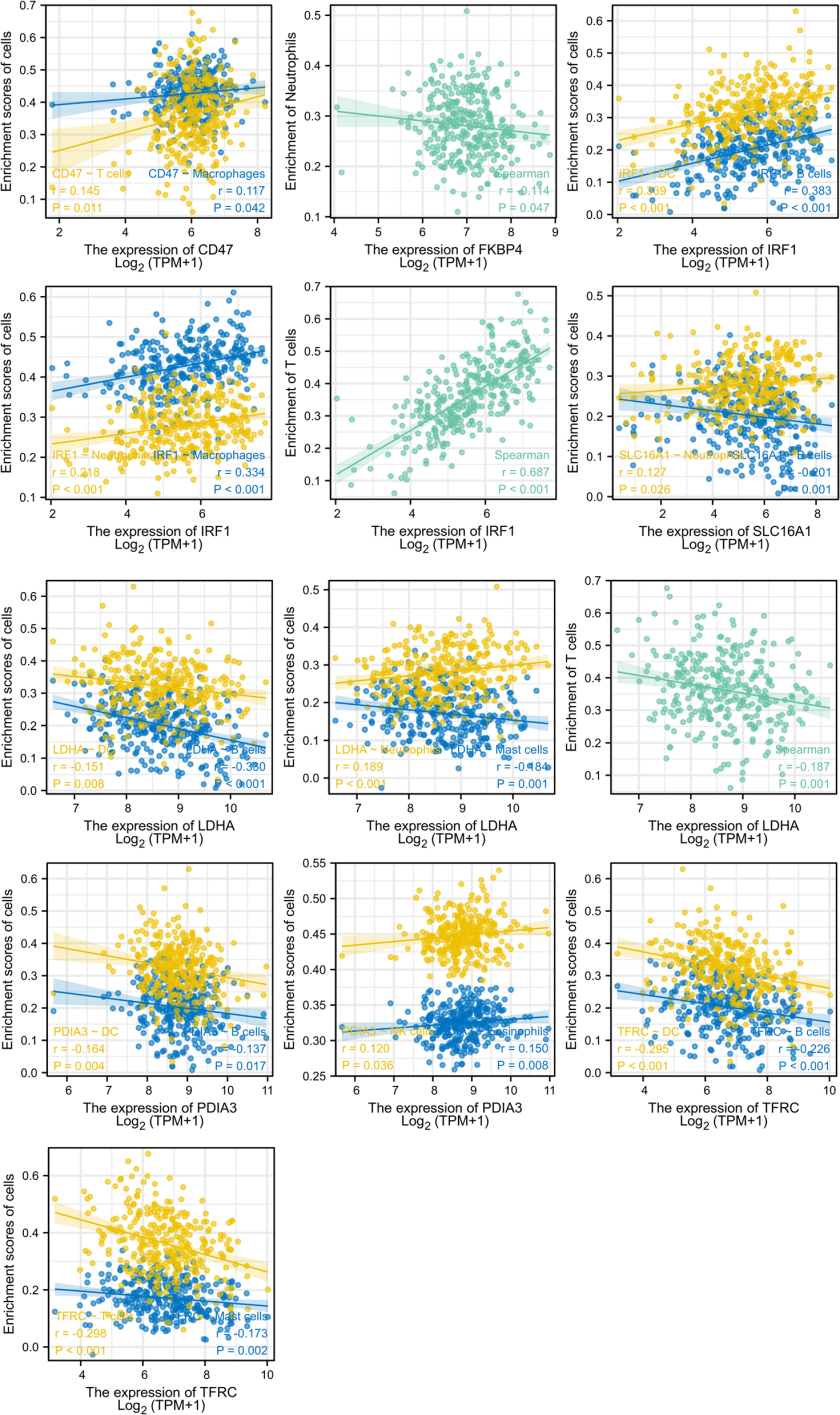
Immunoinfiltration of seven genes in SCC

Immunization is closely related to tumor regression. Therefore, we further investigated whether these seven genes were associated with the expression of immune cells (Fig. 13). CD47 was positively correlated with the number of T cells and macrophages; FKBP4 was negatively correlated with the number of neutrophils; IRF-1 was positively correlated with the number of dendritic cells, B cells, macrophages, neutrophils, and T cells; LDHA was negatively correlated with the number of dendritic cells, B cells, mast cells, and T cells, and positively correlated with the number of neutrophils; PDIA3 was positively correlated with the number of natural killer cells and eosinophils, and negatively correlated with the number of dendritic cells and B cells; TFRC was negatively correlated with the number of dendritic cells, B cells, T cells, and mast cell; and SLC16A1 was positively correlated with the number of neutrophils and negatively correlated with the number of B cells.

### Four mRNAs and proteins were underexpressed in sh_hsa_circ_0000276 cells

**Fig. 14:**
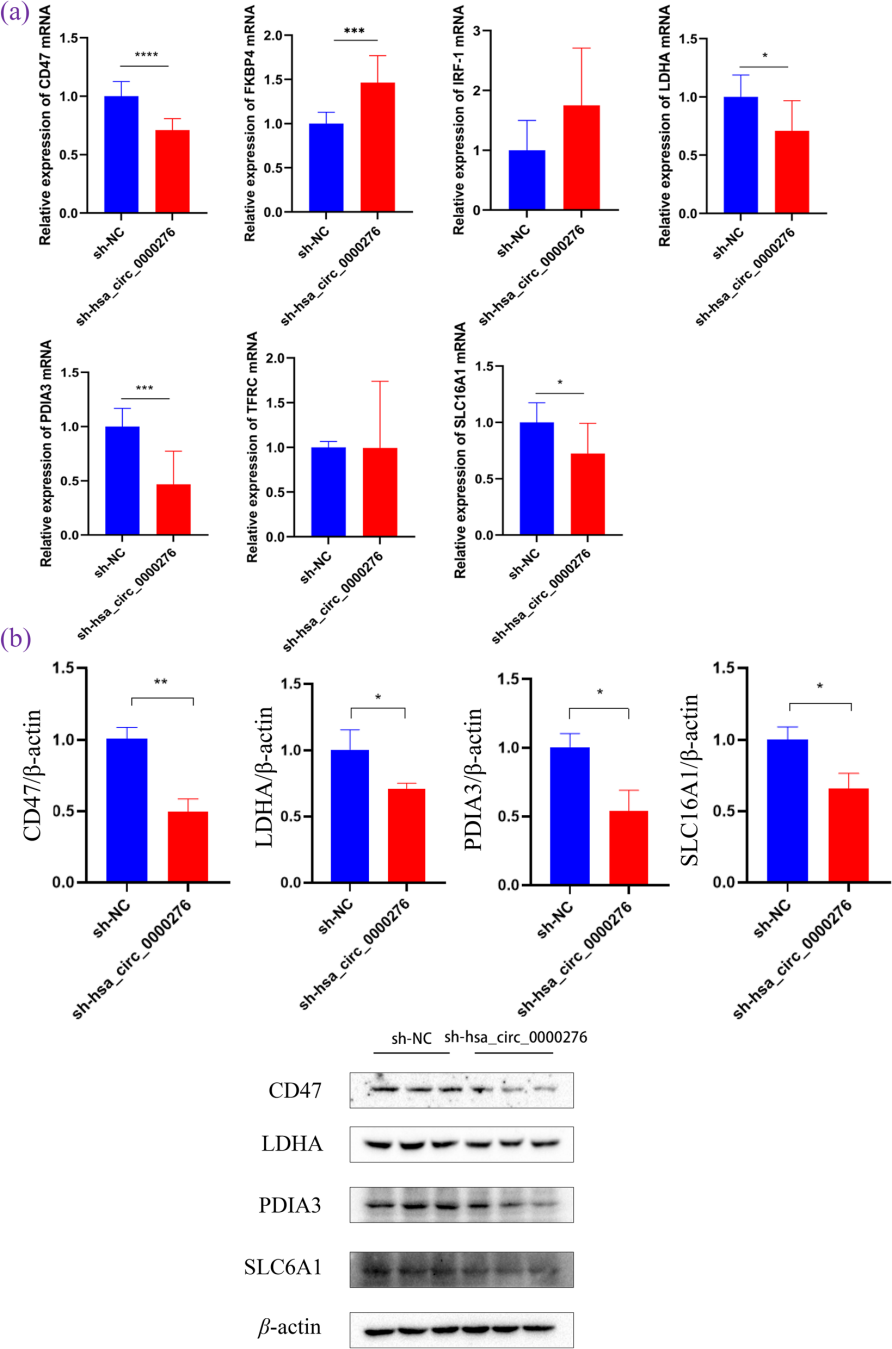
Verification of the predicted gene. **a** qRT-PCR showed the expression of CD47, FKBP4, IRF-1, LDHA, PDIA3, TFRC, and SLC16A1 mRNAs. **b** Western blotting showed the expression of CD47, LDHA, PDIA3, and SLC16A1 proteins. Data are presented as the mean ± SEM (*n* = 3 per group). *p < 0.05, **p < 0.01, *** *p* < 0.001

Next, we detected the expression of each molecule in the sh_hsa_circ_0000276 cell model. The mRNA levels of CD47, LDHA, PDIA3, and SLC16A1 were lower than those of the control group, which was consistent with our prediction. However, those of FKBP4, IRF-1, and TFRC were either contrary to expectations or had an unclear trend; therefore, we determined their protein levels by Western blotting and confirmed results in accordance with the trend of mRNA levels. Original blots are presented in Supplementary Fig. 1 (Fig. 14).

## Discussion

In China, three ways have been adopted to prevent and treat cervical cancer: vaccination, screening, and treatment. In response to the Global Strategy for Accelerating the Elimination of Cervical Cancer released by the WHO, efforts for vaccination and screening have intensified; however, cervical cancer remains the most common malignant tumor in women. According to the pathological classification, SCC accounts for 85–90% of all cervical cancer types and is characterized by a high recurrence rate, strong metastatic nature, and poor prognosis [19]. The ceRNA network has become a research hotspot in recent years to validate confirmed genes, allowing a better understanding of their molecular effects on cervical cancer. Therefore, by exploring the ceRNA network in cervical cancer, this study aimed to identify novel molecular markers to treat cervical cancer and predict its prognosis.

With the development of high-throughput microarray technology, many ncRNAs have been identified; however, they lack protein coding functions. For example, miRNAs are a class of well-characterized ncRNAs that use complementary pairing to regulate post-transcriptional target gene expression. circRNAs are a recent research hotspot in the ncRNA field and are enriched in miRNA binding sites. In addition, ncRNAs play different roles in various cancer-related processes, including proliferation, invasion, metastasis, and metabolism. In the context of ncRNAs in cancer, the ceRNA hypothesis states that when RNA transcripts share the same MRE, ceRNA molecules, including circRNA and competitive mRNAs, can compete for miRNA binding, thus, regulating each other's expression [20].

In this study, after verifying that hsa_circ_0000276 possesses a binding site for miR-154-5p, we collected fresh tissue samples from several cases and found that hsa_circ_0000276 expression was upregulated in LSIL, HSIL, and SCC tissues compared to that in healthy tissues using qRT-PCR, as well as in cells. Uncontrolled continuous proliferation and immortalization of cancer cells promote the progression of cervical cancer. Therefore, we conducted experiments on biological functions related to proliferation, cell cycle, and apoptosis of cancer cells and found that silencing hsa_circ_0000276 in vitro inhibited the proliferation of cervical cancer cells, regulated the cell cycle, and promoted apoptosis, indicating that hsa_circ_0000276 is an important regulatory factor for the growth of cervical cancer cells. In conclusion, we found that hsa_circ_0000276 was aberrantly expressed in the progression of cervicitis to cervical cancer, promoted G1/S transition and cell proliferation, inhibited apoptosis in cervical squamous carcinoma cells, and played an important role as a pro-cancer factor in cervical carcinogenesis.

**Fig. 15:**
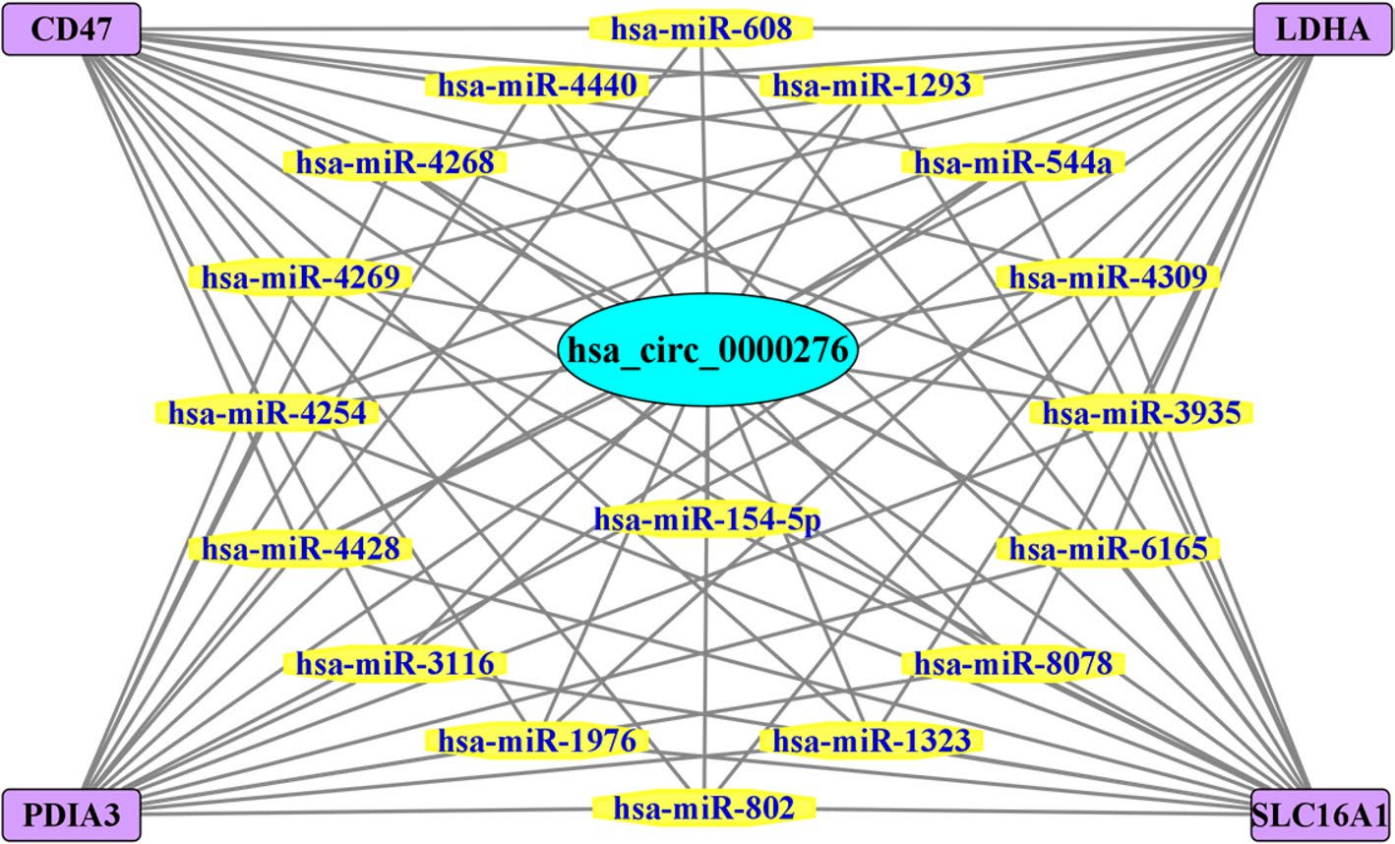
ceRNA regulatory network

After identifying hsa_circ_0000276 as the central molecule in the ceRNA network of HPV16-positive cervical squamous carcinoma, samples from cancerous and paracancerous tissues were used to screen the relevant miRNAs and their downstream mRNAs by differential expression levels and expression trends. DEM analysis using GO and KEGG databases revealed that DEMs in the network were associated with multiple BPs involving RNA, including translation initiation, ribosomes, RNA transport, and degradation; among these, ribosome-related genes are critical for protein formation, cell cycle regulation, and p53 activation, indicating that they can cause cancer transformation [21]. The seven most critical mRNAs were targeted, all of which were associated with immune infiltration, suggesting that hsa_circ_0000276 may be involved in the development and progression of cervical cancer by regulating immune infiltration. After experimental verification, the mRNA and protein levels of CD47, LDHA, PDIA3, and SCL16A1 were confirmed to be downregulated in sh_hsa_circ_0000276 cells. Finally, a ceRNA network comprising 17 miRNAs and 4 mRNAs centered on hsa_circ_0000276 was constructed by evaluating the prognosis and validating the expression trends (Fig. 15).

CD47, also known as integrin-related protein, is a transmembrane protein belonging to the immunoglobulin superfamily that binds to the inhibitory immune receptor signal regulatory protein α, superficially on macrophages and inhibits their phagocytosis [22]. CD47 is overexpressed on the surface of cells of many cancer types; as such, blocking CD47 can treat the progression of these cancers. Xu et al. [23] found that inhibiting lysine-specific demethylase 1 reduced the expression of CD47 and PD-L1 in cervical cancer subcutaneous transplants and that blocking CD47/PD-L1 enhanced its therapeutic effect. CD47 affected the phagocytosis of macrophages, and its binding to SIRPγ affected T cells [24]. Our results demonstrated an association between CD47 and cervical cancer as evidenced by the reduced number of macrophages and T cells.

Lactate dehydrogenase (LDHA) is a key enzyme in the glycolytic process, and the acidic environment established by glycolysis contributes to the immune escape of cancer cells. Inhibition of LDHA reduces the proliferation of many cancer cells, such as those in kidney [25], breast [26], and bladder cancers [27]. In cervical cancer, downregulation of phosphorylated stress-induced phosphoprotein 1 inhibits the progression of cervical cancer by reducing glycolysis and decreasing the level of LDHA [28]. Zhang et al. [29] reduced the proliferation of cervical cancer cells by inhibiting LDHA, and their results are consistent with our bioinformatics results. In terms of immune cells, lactate produced by glycolysis promoted the transformation of pro-inflammatory and anticancer M1 macrophages to anti-inflammatory and pro-cancer M2 macrophages through epigenetic modification. In this study, LDHA showed multiple correlations with immune invasion of cervical cancer.

Protein disulfide isomerase (PDI) A3 (PDIA3) is a crucial member of the PDI family and has recently attracted attention for its effect on human cancer owing to its broad significance in disease development. PDIA3 regulates the proliferation, invasion, and migration of many tumors, and PDIA3 knockdown significantly inhibits the proliferation, invasion, and migration ability of multidrug-resistant gastric cancer cells [30]. In TCGA and Chinese Glioma Genome Atlas datasets, PDIA3 is highly correlated with various tumors, and its overexpression has been associated with a poorer prognosis in cervical cancer. Moreover, PDIA3 is involved in suppressing antitumor immunity through multiple immune modulations. Zhang et al. [31] found that PDIA3 expression in gliomas is correlated with a variety of infiltrating immune cells, including those responsible for antitumor responses as well as immunosuppressive cells. However, in our study, PDIA3 was associated with fewer dendritic and B cells, suggesting that it may be involved in immune infiltration of the cervical cancer microenvironment.

The solute carrier family 16 member 1 (SLC16A1) belongs to the proton-coupled monocarboxylate transporter protein family and is a key transcription factor regulating cancer progression and metastasis [32]. The high expression of SLC16A1 in the urinary system has been associated with a poor prognosis and abnormal epigenetic processes in patients with urological tumors [33]. SLC16A1 expression is significantly higher in high-grade gliomas than in non-tumor controls and low-grade gliomas, and patients with high SLC16A1 expression have a poor prognosis [34]. SLC16A1 is highly expressed in cervical cancer and has been associated with the infiltration of neutrophils and B cells, suggesting its involvement in the progression and immune infiltration of cervical cancer.

In recent years, developments in bioinformatics technology have identified a series of circRNAs related to various malignancies. However, understanding of biological functions of circRNAs in cervical cancer progression is still in the initial stages, and the mechanisms by which circRNAs influence cervical cancer development and metastasis have not been elucidated. Determining the role of circRNA in cervical carcinogenesis cannot be based on predictive analysis alone; thus, further validation using cellular and molecular biology experiments is required.

## Supplementary Information

**Additional file 1.** Information on the 28 circRNAs.

## Data Availability

All data generated or analyzed during this study are included in this published article.

## Abbreviations

AUC: Area under the receiver operating characteristic curve
BP: Biological process
CCK-8: Cell Counting Kit
CI: Confidence interval
circRNA: Circular RNA
Ct: Cycle threshold
DE: Differentially expressed
FC: Fold change
FKBP4: FK506 binding protein 4
GEPIA: Gene Expression Profiling Interactive Analysis
GO: Gene Ontology
HSIL: High-grade squamous intraepithelial lesion
IRF-1: Interferon regulatory factor 1
KEGG: Kyoto Encyclopedia of Genes and Genomes
LDHA: Lactate dehydrogenase A
lncRNA: Long non-coding RNA
LSIL: Low-grade squamous intraepithelial lesion
miRNA: MicroRNA
MRE: MiRNA response element
MUT: Mutant
ncRNA: Non-coding RNA
NF-κB: Nuclear factor kappa B
PC: Positive control
PCR: Polymerase chain reaction
PD: Programmed cell death
PD-L1: Programmed cell death ligand 1
PDI: Protein disulfide isomerase
PDIA3: Protein disulfide isomerase A3
PLAGL2: PLAG1-like zinc finger 2
PPI: Protein–protein interaction
RMA: Robust multiarray analysis
SCC: Squamous cell carcinoma
shRNA: Short hairpin RNA
SLC16A1: Solute carrier family 16 member 1
TCGA: The Cancer Genome Atlas
TFRC: Transferrin receptor protein 1
WT: Wild type
