# Supplementary material for: The hsa_circ_0000276-ceRNA regulatory network and immune infiltration in cervical cancer
Source: BMC Cancer. 2023 Mar 9;23:222. doi: 10.1186/s12885-023-10636-5 (PMC9999601; doi:10.1186/s12885-023-10636-5)
Supplement: Supplementary file 1 — Additional file 1. Information on the 28 circRNAs. [file 12885_2023_10636_MOESM1_ESM.docx]

Additional Files

Additional file 1 — Information on the 28 circRNAs

| **circRNA** | **FC** | **Scores** | **Energy** | **Start** | **End** | **URT binding length** | **binding illustration** |
| --- | --- | --- | --- | --- | --- | --- | --- |
| hsa_circ_0000276 | 1.362059395 | 167 | -27.76 | 5326 | 5350 | 22 | \|\|\|\|\|\| \| \| \|\|\|\|\|\|\|\|\| |
| hsa_circ_0000276 | 1.362059395 | 167 | -27.76 | 11798 | 11822 | 22 | \|\|\|\|\|\| \| \| \|\|\|\|\|\|\|\|\| |
| hsa_circ_0068781 | 1.964121127 | 160 | -16.52 | 13438 | 13461 | 18 | \|\|\|\| :\|\| \|\|\|\|\|\|\|\| |
| hsa_circ_0068781 | 1.964121127 | 160 | -16.52 | 43197 | 43220 | 18 | \|\|\|\| :\|\| \|\|\|\|\|\|\|\| |
| hsa_circ_0084647 | 2.316516844 | 160 | -20.98 | 1720 | 1741 | 19 | :\|\|\| \|\| :\| \|\|\|\|\|\|\| |
| hsa_circ_0084647 | 2.316516844 | 160 | -20.98 | 4689 | 4710 | 19 | :\|\|\| \|\| :\| \|\|\|\|\|\|\| |
| hsa_circ_0043110 | 2.744452693 | 159 | -18.1 | 881 | 903 | 16 | \|\|\|\|: \| \|\|\|\|\|\|\|\| |
| hsa_circ_0043110 | 2.744452693 | 159 | -18.1 | 1969 | 1991 | 16 | \|\|\|\|: \| \|\|\|\|\|\|\|\| |
| hsa_circ_0071623 | 1.724627567 | 158 | -13.75 | 914 | 936 | 19 | \| \|\|\|: \|\| \| \|\|\|\|\|\|\| |
| hsa_circ_0071623 | 1.724627567 | 158 | -13.75 | 2113 | 2135 | 19 | \| \|\|\|: \|\| \| \|\|\|\|\|\|\| |
| hsa_circ_0032076 | 2.589904305 | 155 | -15.51 | 565 | 587 | 20 | \|\|\| :\| : :\| \|\|\|\|\|\|\|\| |
| hsa_circ_0032076 | 2.589904305 | 155 | -15.51 | 2751 | 2773 | 20 | \|\|\| :\| : :\| \|\|\|\|\|\|\|\| |
| hsa_circ_0072652 | 2.132105181 | 154 | -13.84 | 1835 | 1855 | 15 | \|\|\| \|: \|\|\|\|\|\|\|\| |
| hsa_circ_0072652 | 2.132105181 | 154 | -13.84 | 8523 | 8543 | 15 | \|\|\| \|: \|\|\|\|\|\|\|\| |
| hsa_circ_0090386 | 1.782549121 | 154 | -19.22 | 1337 | 1357 | 19 | \|: \|\|\|:\|\| \|\|\|\|\|\|\| |
| hsa_circ_0090386 | 1.782549121 | 154 | -19.22 | 4936 | 4956 | 19 | \|: \|\|\|:\|\| \|\|\|\|\|\|\| |
| hsa_circ_0020981 | 4.424975715 | 154 | -22.75 | 200 | 225 | 23 | :\|\|\|: \|\|:: :\|\|\|\|\|\|\|\|\| |
| hsa_circ_0020981 | 4.424975715 | 154 | -22.75 | 527 | 552 | 23 | :\|\|\|: \|\|:: :\|\|\|\|\|\|\|\|\| |
| hsa_circ_0091564 | 3.186070973 | 153 | -12.51 | 1082 | 1103 | 16 | \|:\|\| : \|\|\|\|\|\|\|\| |
| hsa_circ_0091564 | 3.186070973 | 153 | -12.51 | 4769 | 4790 | 16 | \|:\|\| : \|\|\|\|\|\|\|\| |
| hsa_circ_0018228 | 2.677920565 | 151 | -17.01 | 4091 | 4113 | 20 | :\| \|\|\|:: \|: \|\|\|\|\|\|\| |
| hsa_circ_0018228 | 2.677920565 | 151 | -17.01 | 9684 | 9706 | 20 | :\| \|\|\|:: \|: \|\|\|\|\|\|\| |
| hsa_circ_0018458 | 1.481121713 | 150 | -10.87 | 1621 | 1642 | 13 | \|:\| \|\|\|\|\|\|\|\| |
| hsa_circ_0018458 | 1.481121713 | 150 | -10.87 | 3517 | 3538 | 13 | \|:\| \|\|\|\|\|\|\|\| |
| hsa_circ_0034354 | 3.537030837 | 149 | -16.07 | 2232 | 2256 | 20 | \|\|::\|:: \| \|\|\|\|\|\|\|\| |
| hsa_circ_0034354 | 3.537030837 | 149 | -16.07 | 6218 | 6242 | 20 | \|\|::\|:: \| \|\|\|\|\|\|\|\| |
| hsa_circ_0032076 | 2.589904305 | 148 | -12.45 | 161 | 182 | 19 | \|\| \|\|\| : \|\|\|\|\|\|\| |
| hsa_circ_0032076 | 2.589904305 | 148 | -12.45 | 2347 | 2368 | 19 | \|\| \|\|\| : \|\|\|\|\|\|\| |
| hsa_circ_0068781 | 1.964121127 | 147 | -12.68 | 12130 | 12151 | 14 | \|:\| \| \|\|\|\|\|\|\| |
| hsa_circ_0068781 | 1.964121127 | 147 | -12.68 | 41889 | 41910 | 14 | \|:\| \| \|\|\|\|\|\|\| |
| hsa_circ_0004671 | 2.630392498 | 147 | -10.29 | 38 | 59 | 10 | \| \|\|\|\|\|\|\|\| |
| hsa_circ_0004671 | 2.630392498 | 147 | -10.29 | 863 | 884 | 10 | \| \|\|\|\|\|\|\|\| |
| hsa_circ_0091411 | 3.566262081 | 147 | -9.35 | 1267 | 1288 | 14 | \|\|:\| \|\|\|\|\|\|\| |
| hsa_circ_0091411 | 3.566262081 | 147 | -9.35 | 2557 | 2578 | 14 | \|\|:\| \|\|\|\|\|\|\| |
| hsa_circ_0006373 | 2.612787974 | 146 | -13.44 | 261 | 279 | 17 | \|\| \|\|:\| \|\|\|\|\|\|\| |
| hsa_circ_0006373 | 2.612787974 | 146 | -13.44 | 834 | 852 | 17 | \|\| \|\|:\| \|\|\|\|\|\|\| |
| hsa_circ_0075892 | 1.995223901 | 146 | -9.83 | 1345 | 1366 | 17 | \|\|: : : \| \|\|\|\|\|\|\| |
| hsa_circ_0075892 | 1.995223901 | 146 | -9.83 | 3140 | 3161 | 17 | \|\|: : : \| \|\|\|\|\|\|\| |
| hsa_circ_0061761 | 2.400613825 | 146 | -10.29 | 1261 | 1281 | 19 | \|\| ::\| \|\| \|\|\|\|\|\|\| |
| hsa_circ_0061761 | 2.400613825 | 146 | -10.29 | 2724 | 2744 | 19 | \|\| ::\| \|\| \|\|\|\|\|\|\| |
| hsa_circ_0016216 | 1.682730101 | 145 | -13.77 | 1440 | 1459 | 19 | \| \|\|::\|: : \|\|\|\|\|\|\| |
| hsa_circ_0016216 | 1.682730101 | 145 | -13.77 | 3908 | 3927 | 19 | \| \|\|::\|: : \|\|\|\|\|\|\| |
| hsa_circ_0072652 | 2.132105181 | 145 | -12.67 | 5756 | 5777 | 8 | \|\|\|\|\|\|\|\| |
| hsa_circ_0072652 | 2.132105181 | 145 | -12.67 | 12444 | 12465 | 8 | \|\|\|\|\|\|\|\| |
| hsa_circ_0074755 | 2.431589019 | 145 | -10.71 | 986 | 1007 | 8 | \|\|\|\|\|\|\|\| |
| hsa_circ_0074755 | 2.431589019 | 145 | -10.71 | 1995 | 2016 | 8 | \|\|\|\|\|\|\|\| |
| hsa_circ_0018794 | 1.850181416 | 145 | -14.31 | 1244 | 1265 | 16 | \| ::\| \| \|\|\|\|\|\|\| |
| hsa_circ_0018794 | 1.850181416 | 145 | -14.31 | 3393 | 3414 | 16 | \| ::\| \| \|\|\|\|\|\|\| |
| hsa_circ_0032214 | 1.806519248 | 145 | -12.97 | 6303 | 6319 | 19 | \|\|\|\| \|\|\| \|\|\|\|\|\|\| |
| hsa_circ_0032214 | 1.806519248 | 145 | -12.97 | 13635 | 13651 | 19 | \|\|\|\| \|\|\| \|\|\|\|\|\|\| |
| hsa_circ_0084493 | 2.785644479 | 144 | -14 | 1489 | 1510 | 21 | \|\|\| \|\|\| :\| \|\|\|\|\|\|\| |
| hsa_circ_0084493 | 2.785644479 | 144 | -14 | 7764 | 7785 | 21 | \|\|\| \|\|\| :\| \|\|\|\|\|\|\| |
| hsa_circ_0067712 | 2.336163264 | 143 | -17.77 | 1611 | 1633 | 20 | :\|\| \| \|\|:\|\| \|\|\|\|:\|\| |
| hsa_circ_0067712 | 2.336163264 | 143 | -17.77 | 4860 | 4882 | 20 | :\|\| \| \|\|:\|\| \|\|\|\|:\|\| |
| hsa_circ_0072652 | 2.132105181 | 143 | -12.09 | 2080 | 2103 | 17 | \|\|\|\| \| \|\|\|\|\|\|\| |
| hsa_circ_0072652 | 2.132105181 | 143 | -12.09 | 8768 | 8791 | 17 | \|\|\|\| \| \|\|\|\|\|\|\| |
| hsa_circ_0075892 | 1.995223901 | 143 | -24.35 | 1071 | 1093 | 20 | \|:\|\|\|\|\|\|\|:\| :\|\|\|\|:\| |
| hsa_circ_0075892 | 1.995223901 | 143 | -24.35 | 2866 | 2888 | 20 | \|:\|\|\|\|\|\|\|:\| :\|\|\|\|:\| |
| hsa_circ_0061535 | 2.354321143 | 143 | -17.77 | 5773 | 5794 | 17 | \| \|\| \|\| \|\|\|\|\|\|\|\| |
| hsa_circ_0061535 | 2.354321143 | 143 | -17.77 | 12495 | 12516 | 17 | \| \|\| \|\| \|\|\|\|\|\|\|\| |
| hsa_circ_0061761 | 2.400613825 | 143 | -19.38 | 381 | 401 | 15 | \|:\|\| \|:\|\|\|\|\|\|\|\| |
| hsa_circ_0061761 | 2.400613825 | 143 | -19.38 | 1844 | 1864 | 15 | \|:\|\| \|:\|\|\|\|\|\|\|\| |
| hsa_circ_0011661 | 2.363921141 | 142 | -9.01 | 222 | 243 | 13 | \|: \| \|\|\|\|\|\|\| |
| hsa_circ_0011661 | 2.363921141 | 142 | -9.01 | 973 | 994 | 13 | \|: \| \|\|\|\|\|\|\| |
| hsa_circ_0072394 | 1.799280743 | 141 | -11.41 | 138 | 157 | 15 | \|\|::\| \|\|\|\|\|\|\| |
| hsa_circ_0072394 | 1.799280743 | 141 | -11.41 | 789 | 808 | 15 | \|\|::\| \|\|\|\|\|\|\| |
| hsa_circ_0032214 | 1.806519248 | 141 | -12.67 | 2329 | 2350 | 12 | \|\|\| \|\|\|\|\|\|:\| |
| hsa_circ_0032214 | 1.806519248 | 141 | -12.67 | 9661 | 9682 | 12 | \|\|\| \|\|\|\|\|\|:\| |
| hsa_circ_0018228 | 2.677920565 | 140 | -10.65 | 2557 | 2578 | 7 | \|\|\|\|\|\|\| |
| hsa_circ_0018228 | 2.677920565 | 140 | -10.65 | 8150 | 8171 | 7 | \|\|\|\|\|\|\| |
| hsa_circ_0003821 | 1.665537284 | 140 | -13.78 | 383 | 404 | 7 | \|\|\|\|\|\|\| |
| hsa_circ_0003821 | 1.665537284 | 140 | -13.78 | 2212 | 2233 | 7 | \|\|\|\|\|\|\| |
| hsa_circ_0061535 | 2.354321143 | 140 | -8.11 | 5050 | 5071 | 7 | \|\|\|\|\|\|\| |
| hsa_circ_0061535 | 2.354321143 | 140 | -8.11 | 11772 | 11793 | 7 | \|\|\|\|\|\|\| |
